# Herpes Simplex Virus 1 UL2 Inhibits the TNF-α–Mediated NF-κB Activity by Interacting With p65/p50

**DOI:** 10.3389/fimmu.2020.00549

**Published:** 2020-05-13

**Authors:** Mingsheng Cai, Zongmin Liao, Xingmei Zou, Zuo Xu, Yuanfang Wang, Tong Li, Yiwen Li, Xiaowen Ou, Yangxi Deng, Yingjie Guo, Tao Peng, Meili Li

**Affiliations:** ^1^Guangdong Provincial Key Laboratory of Allergy and Clinical Immunology, The Second Affiliated Hospital of Guangzhou Medical University, Sino-French Hoffmann Institute, School of Basic Medical Science, Guangzhou Medical University, Guangzhou, China; ^2^Department of Scientific Research and Education, Yuebei People’s Hospital, Shaoguan, China; ^3^State Key Laboratory of Respiratory Diseases, Sino-French Hoffmann Institute, Guangzhou Medical University, Guangzhou, China; ^4^South China Vaccine Corporation Limited, Guangzhou Science Park, Guangzhou, China

**Keywords:** innate immunity, HSV-1, UL2, NF-κB, IL-8

## Abstract

Herpes simplex virus 1 (HSV-1) is a large double-stranded DNA virus that encodes at least 80 viral proteins, many of which are involved in the virus–host interaction and are beneficial to the viral survival and reproduction. However, the biological functions of some HSV-1–encoded proteins are not fully understood. Nuclear factor κB (NF-κB) activation is the major antiviral innate response, which can be triggered by various signals induced by cellular receptors from different pathways. Here, we demonstrated that HSV-1 UL2 protein could antagonize the tumor necrosis factor α (TNF-α)–mediated NF-κB activation. Co-immunoprecipitation assays showed that UL2 could interact with the NF-κB subunits p65 and p50, which also revealed the region of amino acids 9 to 17 of UL2 could suppress the NF-κB activation and interact with p65 and p50, and UL2 bound to the immunoglobulin-like plexin transcription factor functional domain of p65. However, UL2 did not affect the formation of p65/p50 dimerization and their nuclear localizations. Yet, UL2 was demonstrated to inhibit the NF-κB activity by attenuating TNF-α–induced p65 phosphorylation at Ser536 and therefore decreasing the expression of downstream inflammatory chemokine interleukin 8. Taken together, the attenuation of NF-κB activation by UL2 may contribute to the escape of host’s antiviral innate immunity for HSV-1 during its infection.

## Introduction

Herpes simplex virus 1 (HSV-1) is an important human pathogen, which is carried with high frequencies in the world with its well-known ability to establish a lifelong latent infection in neurons and trigger a reactivation and lytic infection mainly in human epithelial or mucosal cells (1). Herpes simplex virus 1 infection can lead to many diseases, such as keratoconjunctivitis and oropharynx and genital ulcers, accompanied with clinical manifestations of pain, fever, and swollen lymph nodes, which are responsible for its severe morbidity (2–4). Therefore, HSV-1 has brought great harm to the health of worldwide population.

UL2 protein, a uracil DNA glycosylase (UDG) that is the early (E) gene product of HSV-1 *UL2* (5), is involved in the base excision repair pathway, which removes uracil by cleaving the *N*-glycosidic bond between uracil and deoxyribose backbone, leaving an apyrimidinic site to reduce the point mutations and strand breakage that are associated with the presence of uracil in DNA replication (6–8). Moreover, UL2, which is localized in the nucleus under the help of some subcellular transport receptors (8–10), is shown to be related to the viral replisome via its interaction with UL30 (5, 9). Although UDG may be dispensable for the viral replication in culture cells, UDG mutant exhibits reduced neurovirulence, and a decreased frequency of reactivation from latency, indicating that UDG may be vital for HSV-1 reactivation in quiescent neuronal cells, during which the genome might accumulate uracil as a result of spontaneous deamination of cytosine (5, 11). Besides, other herpesviral UDGs are shown to be required for the effective viral gene expression and DNA synthesis, as well as efficient viral production *in vivo* (12–14). Nevertheless, the enzyme activity of UL2 can be inhibited by its corresponding inhibitor 6-(4-anilinoalkyl)-uracil or *Staphylococcus aureus* UDG (6, 15). Accordingly, UL2 may play a significant role in the virulence, latency, and reactivation of HSV-1 infection (11, 16–18). However, the exact roles of UL2 during HSV-1 infection are still poorly understood.

Innate immune system is the first line for host defense during viral infection, and the recognition of viral constituents is mediated by diverse pattern recognition receptors, which lead to the activation of many intracellular signaling pathways, followed by the production of a number of interferons (IFNs), and inflammatory cytokines (19–27). Nuclear factor κB (NF-κB) signaling is an important intracellular antiviral pathway. In mammals, the NF-κB family includes five functional proteins: p50, p52, p65 (RelA), RelB, and c-Rel, which are encoded by the genes *NF-KB1* (precursor is p105), *NF-KB2* (precursor is p100), *RELA*, *RELB*, and *REL*, respectively (28). Generally, these five transcription factors are assembled as homodimer or heterodimer. Like RelB/p52 and p65/p50, although some of them have greater affinity due to their structure dimers, combinations of each member are still possible (28, 29).

In the quiescent cells, p65/p50, and p65/p52 dimers are usually bound to the inhibitory molecule of IκB family proteins to form a complex (in most cases, IκBα), which prevents them to transfer into the nucleus (29). Therefore, the NF-κB complex presents as a transcriptionally inactive state in the cytoplasm. However, extensive stimulation such as cytokines, microbial and viral products, DNA damage, and oxidative stress, which can be recognized by many proinflammatory cytokine receptors, including Toll-like receptor family members, interleukin 1 (IL-1) receptor, and tumor necrosis factor receptor (TNFR), can significantly activate NF-κB signaling that has a similar signal transduction cascade substantially produced by phosphate transfer (28, 30–34).

When TNFR is stimulated by TNF-α, TNFR will recruit the adaptor protein TNF receptor–associated death domain (TRADD), and then TRADD activates TNFR-associated factor 2 (TRAF2) and the downstream receptor-interacting protein 1 (RIP1). The function of activated TRAF2 is to mediate the K63-linked RIP1 polyubiquitination. Subsequently, ubiquitinated RIP1 further recruits TGF-β–activated kinase 1 (TAK1) and at last activates the IκB kinase (IKK), resulting in the succedent phosphorylation and ubiquitination degradation of IκBα mediated by 26S proteasome. Finally, NF-κB dimers can be released from IκBα and translocated into the nucleus, leading NF-κB to bind to specific promoters and induce target gene expressions (30, 35, 36).

Viral infection can significantly activate the classical NF-κB pathway; this process promotes the expression of various IFNs and inflammatory cytokines, such as IFN-α, IFN-β, IL-6, IL-8, and TNF-α, which in turn reduce and inhibit viral replication and proliferation. Hence, many viruses have evolved different strategies to inhibit NF-κB signaling for their replications and to prevent virus-induced apoptosis. For example, orf virus virion-associated protein 119 is reported to inhibit IKK complex activation and target the retinoblastoma protein to inhibit NF-κB signaling in the early infection (37). Influenza A virus–encoded virulence factor protein NS1 is shown to crumble IKK function to counteract host NF-κB–mediated antiviral response (38). Herpes simplex virus 1 also can disturb the NF-κB pathway by encoding some important proteins to execute this function. Herpes simplex virus 1 UL36, a ubiquitin-specific protease, abrogates NF-κB activation by cleaving polyubiquitin chains of IκBα and therefore restricts proteasome-dependent degradation of IκBα (39). Another protein kinase HSV-1 US3 hyperphosphorylates p65/RelA at the site of Ser75 and consequently dampens NF-κB activation (40). In our study, it is the first time to demonstrate that HSV-1 UL2 could antagonize NF-κB activity and inhibit downstream inflammatory cytokine IL-8 expression, by attenuating TNF-α–induced p65 phosphorylation at Ser536, which may help HSV-1 to evade host’s antiviral innate immunity.

## Materials and Methods

### Viruses and Cytokine

Wild-type (WT) HSV-1 BAC GFP Luc (F strain, simultaneously expressing firefly luciferase and GFP tag) was provided by Dr. Chunfu Zheng (Fujian Medical University), which was propagated and preserved in our laboratory. The detailed construction procedure and identification of UL2-related recombinant viruses, including UL2 deletion (Del) and UL2 revertant (Rev) HSV-1 BAC GFP Luc, were specifically described and successfully constructed using homologous recombination technology in our recent study (41). Recombinant human TNF-α was purchased from Peprotech.

### Cells

Human embryonic kidney (HEK) 293T and HeLa cells were grown in Dulbecco modified MEM (DMEM; Gibco-BRL, Grand Island, NY, United States) with 10% heat inactivated fetal bovine serum (FBS; Gibco-BRL, Grand Island, NY, United States) at the temperature 37°C and in a humidified 5% CO_2_ incubator (Thermo, Fisher Scientific, Waltham, MA, United States).

### Antibodies

Anti-Flag (DYKDDDDK) (3B9), anti-Myc (19C2), and anti-hemagglutinin (HA) (26D11) mouse monoclonal antibodies (mAbs) were obtained from ABmart. Anti–β-actin, anti-EYFP, anti-EGFP, anti-p65, and anti-p50 polyclonal antibodies were purchased from Proteintech (Rosemont, IL, United States). Fluorescein isothiocyanate (FITC)–conjugated donkey anti–mouse immunoglobulin G (IgG) and Cy5-conjugated goat anti–rabbit IgG were bought from BBI Life Sciences (Shanghai, China). Mouse non-specific IgG Ab was offered by eBioscience (San Diego, CA, United States). The Abs phospho-NF-κB–p65(ser276), phospho-NF-κB–p65(ser536), and alkaline phosphatase (AP) conjugated goat anti–mouse IgG (AP), goat anti–rat IgG (AP), and goat anti–rabbit IgG (AP) were obtained from Affinity Biosciences (Cincinnati, OH, United States). Anti-UL2 pAb was prepared in rat (unpublished data) and stored in our laboratory.

### Plasmids Construction

To construct Flag-tagged UL2 expression plasmid, the open reading frame of HSV-1 (F strain) *UL2* was polymerase chain reaction (PCR) amplified from pYEbac102 (42) with forward primer 5′-CGA AGC TTC GGA ATT CAT GAA GCG GGC CTG CAG CCG and reverse primer 5′-GCA AGC TTA GGA TCC GTA ACC GAC CAG TCG ATG GGT G, and then the purified PCR product was digested with *Eco*RI and *Bam*HI and inserted into the corresponding digested pFlag-N1 vector (regenerated from pEYFP-N1; Clontech, Palo Alto, CA, United States) to yield pUL2-Flag, as described previously (8, 43). pUL2-HA, pUL2-Myc, pUL2-EYFP, and UL2 truncated mutant plasmids bearing EYFP tag and pp65–immunoglobulin-like plexin transcription factor (IPT)–del–Myc were constructed in our laboratory with similar methods. The primers used to construct a series of UL2 truncated mutants fused with EYFP are shown in Table 1. Reporter plasmids NF-κB–Luc and pRL-TK (expressing firefly luciferase and Renilla luciferase, respectively) were kindly gifted from Dr. Zhengli Shi (Wuhan Institute of Virology, Chinese Academy of Sciences). pXP2-pIL-8-Luc was from Dr. Wenlin Huang (38). Pad-N-p65(1-290)-Flag and Pad-N-p65(291-551)-Flag were generous gifts from Dr. Leiliang Zhang (44). pFlag-TRADD, pTRAF2-Flag, pRIP1-Flag, and p65-RHD-Flag were provided by Dr. Chunfu Zheng (39). pFlag-TAK1 and pNIK-Flag were generous gifts from Dr. Jun Cui (School of Life Sciences, Sun Yat-sen University). Other gift plasmids pCMV-Flag-p65 (Prof. Katherine A. Fitzgerald), pFlag-p50 (Dr. Karl-Klaus Conzelmann), pHA-IKKα (Prof. Gangmin Hur), pFlag-IKKβ (Prof. Rao Anjana), pp65-IPT-EGFP (Dr. Masataka Kinjo) (45), and pp65-EYFP (Prof. An Hong) (46) were shown as indicated. All expression plasmids were validated by DNA sequencing.

**TABLE 1:** Primers used for constructing the recombinant HSV-1 UL2 plasmids merged with EYFP.

Plasmids’ name	Primer name	Forward primer (5′→3′)
UL2 (1–334)-EYFP	UL2-HindIII,EcoRI-F	CGAAGCTTCGGAATTCATGAAGCGGGCCTGCAGCCG
	UL2-HindIII,BamHI-R	GCAAGCTTAGGATCCGTAACCGACCAGTCGATGGGTG
UL2 (1–8)-EYFP	UL2 (1–8)-EcoRI-F	AATTCATGAAGCGGGCCTGCAGCCGAAGCTTG
	UL2 (1–8)-BamHI-R	GATCCAAGCTTCGGCTGCAGGCCCGCTTCATG
UL2 (1–17)-EYFP	UL2 (1–17)-EcoRI-F	AATTCATGAAGCGGGCCTGCAGCCGAAGCCCCTCACCACGCCGCCGCCCATCATCGTTG
	UL2 (1–17)-BamHI-R	GATCCAACGATGATGGGCGGCGGCGTGGTGAGGGGCTTCGGCTGCAGGCCCGCTTCATG
UL2 (9–17)-EYFP	UL2 (9–17)-EcoRI-F	AATTCATGCCCTCACCACGCCGCCGCCCATCATCGTTG
	UL2 (9–17)-BamHI-R	GATCCAACGATGATGGGCGGCGGCGTGGTGAGGGCATG
UL2 (1–31)-EYFP	UL2-HindIII,EcoRI-F	CGAAGCTTCGGAATTCATGAAGCGGGCCTGCAGCCG
	UL2 (1–31)-BamHI-R	TTGGATCCAATTTTTGCGGCGGCGTCCCGTC
UL2 (61–75)-EYFP	UL2 (61–75)-EcoRI-F	AATTCATGCGCTCGTCAGGGCCGGCGGGCGCTCCTCGCCGCCCTAGAGGCTGTATG
	UL2 (61–75)-BamHI-R	GATCCATACAGCCTCTAGGGCGGCGAGGAGCGCCCGCCGGCCCTGACGAGCGCATG
UL2 (69–75)-EYFP	UL2 (69–75)-EcoRI-F	AATTCATGCCTCGCCGCCCTAGAGGCTGTTCG
	UL2 (69–75)-BamHI-R	GATCCGAACAGCCTCTAGGGCGGCGAGGCATG
UL2 (69–224)-EYFP	UL2 (69–224)-EcoRI-F	TTGAATTCATGCCTCGCCGCCCTAGAGGCTG
	UL2 (69–224)-BamHI-R	AAGGATCCGTGCAACCGTGGCCGCTCATCCG
UL2 (225–334)-EYFP	UL2 (225–334)-EcoRI-F	TTGAATTCATGCTGGAAAAGTGGGCGCGGGAC
	UL2-HindIII,BamHI-R	GCAAGCTTAGGATCCGTAACCGACCAGTCGATGGGTG
UL2 (225–240)-EYFP	UL2 (225–334)-EcoRI-F	TTGAATTCATGCTGGAAAAGTGGGCGCGGGAC
	UL2 (225–240)-BamHI-R	CGGGATCCAACAGGGTCGTGTTTAGTAACAG
UL2 (225–277)-EYFP	UL2 (225–334)-EcoRI-F	TTGAATTCATGCTGGAAAAGTGGGCGCGGGAC
	UL2 (225–277)-BamHI-R	AAGGATCCAAGAGCATAAACACCAGGCCGGG
UL2 (278–334)-EYFP	UL2 (278–334)-EcoRI-F	TTGAATTCATGTGGGGCGCACATGCCCAGAAT
	UL2-HindIII,BamHI-R	GCAAGCTTAGGATCCGTAACCGACCAGTCGATGGGTG
UL2 (9–17) del -EYFP	UL2 (9–17) del -EcoRI-F	TTGAATTCTATGAAGCGGGCCTGCAGCCGAAGCCCACGTTGGACCCCACCC
	UL2-HindIII,BamHI-R	GCAAGCTTAGGATCCGTAACCGACCAGTCGATGGGTG
UL2 (9–17) (69–75) del -EYFP	UL2 (9–17) del -EcoRI-F	TTGAATTCTATGAAGCGGGCCTGCAGCCGAAGCCCACGTTGGACCCCACCC
	UL2 (69–75) del-R	GAAAACGTCACACCAGCGGGAGCGCCCGCCGGCCCTGACGAG
	UL2 (69–75) del-F	CCCGCTGGTGTGACGTTTTC
	UL2-HindIII,BamHI-R	GCAAGCTTAGGATCCGTAACCGACCAGTCGATGGGTG

### Plasmid Transfection and Dual-Luciferase Reporter Assays

The plasmid transfection and dual-luciferase reporter (DLR) assays were performed as described previously (47). HEK293T cells were plated on 24-well plates (Corning Inc., Corning, NY, United States) at a density of 70 to 80% confluence (2 × 10^5^ cells per well) overnight, and then 100 ng of NF-κB or IL-8 promoter reporter plasmid, 10 ng of pRL-TK (internal control), and the indicated amounts of expression plasmid mixed with polyethylenimine transfection reagent (Polysciences, Warrington, PA, United States) according to the manufacturer’s instructions [PEI (μL):DNA (μg) = 3:1] were cotransfected into cells. Twenty-four hours posttransfection, cells were treated or mock treated with recombinant human TNF-α (10 ng/mL) for 6 h. Then, cell lysates were divided into two aliquots: one aliquot was used for DLR detection, and the other was used for Western blot (WB) analysis to detect the protein expression of transfected plasmid. The transfection efficiency was confirmed using fluorescence microscope when the transfection system contains fluorescence protein-labeled plasmid. And if there was no fluorescence-labeled plasmid in the transfection system, a small amount of non-relevant fluorescent empty vector (such as EYFP vector) was added to confirm the transfection efficiency. Besides, non-relevant empty vector DNA was also added to reach the same amount of transfected DNA for each well. The luciferase activity was assessed using a luciferase assay kit (Promega, Madison, WI, United States). Data were normalized for transfection efficiency through measuring firefly luciferase activity and Renilla luciferase activity, and values were shown as the ratio between firefly and Renilla luciferase. Data were expressed as means ± standard deviations (SDs) from three independent experiments.

### Confocal Microscopy

The confocal microscopy experiments were carried out as described previously (48–50). Briefly, HeLa cells were plated on 24-well plates in DMEM added with 10% FBS at a density of 2 × 10^5^ cells per well overnight before transfection, and then cells were transfected with 500 ng of the indicated plasmids. Twenty-four hours posttransfection, cells were treated with 10 ng/mL of recombinant human TNF-α or mock treated for 30 min, and then the cells were fixed with 4% paraformaldehyde (Beyotime Biotechnology, Shanghai, China) for 30 min at 37°C and incubated in 0.2% Triton X-100 (Beyotime Biotechnology) for 30 min. After that, the cells were probed with the indicated Abs (anti-p65, anti-p50, or anti-HA) for 1.5 h at room temperature, followed by incubation with FITC-conjugated donkey anti–mouse IgG or Cy5-conjugated goat anti–rabbit IgG for 1 h. Finally, the cells were stained with DAPI (4’6-diamidino-2-phe-nylindole) (Cell Signaling Technology, Inc., Danvers, MA, United States) for 3 to 5 min. Images were obtained with a confocal microscope (Axio-Imager-LSM-800; Carl Zeiss, Oberkochen, Germany) using 400 × oil-immersion objective. Each image represented a vast majority of the cells with similar subcellular distribution.

### Viral Infection and Flow Cytometry

HEK293T cells were plated on 12-well plates (Corning) in DMEM supplemented with 10% FBS overnight before infection, and then WT, UL2 Del, or UL2 Rev HSV-1 BAC GFP Luc was dissolved in DMEM medium and added to the cells at an multiplicity of infection (MOI) of 1. The viruses were incubated for 1.5 to 2 h at 37°C in 5% CO_2_ culture incubator and then replaced with medium containing 2% FBS to continue culture for 16 h, and then cells were harvested for flow cytometry analysis, with CytoFLEX (Beckman Coulter, Brea, CA, United States) through 488 single fluorescent channel after gently washing the cells with phosphate-buffered saline (PBS). The data were analyzed using FlowJo software (Tree Star, San Carlos, CA, United States).

### RNA Isolation and Real-Time Quantitative PCR

HEK293T cells or HeLa cells cultured in six-well plates (1.3 × 10^6^ cells per well) were transfected with control vector or UL2 expression plasmid; 24 h posttransfection, cells were treated with TNF-α (10 ng/mL) for 6 h, and total RNA was extracted with TRIzol reagent (Invitrogen, Carlsbad, CA, United States). Samples were then subjected to reverse transcription to cDNA with reverse transcription (RT) reagent (TsingKe Biotechnology, Beijing, China). The acquired cDNA was taken as the template for RT–quantitative PCR (qPCR) to detect the expression of housekeeping gene glyceraldehyde-3-phosphate dehydrogenase (GAPDH) and human IL-8 with qPCR reagent (TsingKe), using qPCR instrument (Bio-Rad, CFX96, Hercules, CA, United States). Primers used for GAPDH (forward primer 5′-AGG TCG GTG TGA ACG GAT TTG and reverse primer 5′-TGT AGA CCA TGT AGT TGA GGT CA) and IL-8 (forward primer 5′-GGT GCA GTT TTG CCA AGG AG and reverse primer 5′-TTC CTT GGG GTC CAG ACA GA) were referred to Tian and colleagues’ (51) and Na Takuathung and colleagues’ (52) reports, respectively. Data were expressed as means ± SD from three independent experiments.

### Co-immunoprecipitation Assays and WB Analysis

The co-immunoprecipitation (Co-IP) and WB were carried out as described previously (8, 53–55). In brief, HEK293T cells were cotransfected with plasmid combinations tagged with Flag, HA, EGFP, EYFP, or Myc. Twenty-four hours posttransfection, cells were harvested and lysed with RIPA lysis buffer (Beyotime Biotechnology) at 4°C for 30 min. When needed, HEK293T cells were infected with WT HSV-1 at an MOI of 1 for 24 h and treated with 10 ng/mL of the TNF-α for an additional 6 h. Then, an equivalent of lysates was incubated with anti-Flag, anti-HA, anti-Myc, anti-p65, anti-p50, or non-specific control mouse antibody (IgG) with 1:1 slurry of protein A/G PLUS-Agarose (Santa Cruz Biotechnology, Dallas, TX, United States) at 4°C overnight. The bead complex was then washed at least three times with PBS. Finally, Co-IPed proteins and cell lysates were subjected to WB analysis with related Abs. All Co-IP/WB assays were repeated at least three times, and similar results were obtained. The original WB results were shown in the section of Supplementary Material.

### Statistical Analysis

Statistical analyses were performed using Student *t* test (unpaired two-tailed *t* test) in GraphPad Prism 6 (GraphPad Software, San Diego, CA, United States) with significant differences marked on the figures. Significance levels were defined as ns, not significant, *P* > 0.05; ^∗^*P* < 0.05; ^∗∗^*P* < 0.01; ^∗∗∗^P < 0.001; and ^****^P < 0.0001.

## Results

### Inhibition of TNF-α–Induced NF-κB Activation by HSV-1 UL2

**FIGURE 1:**
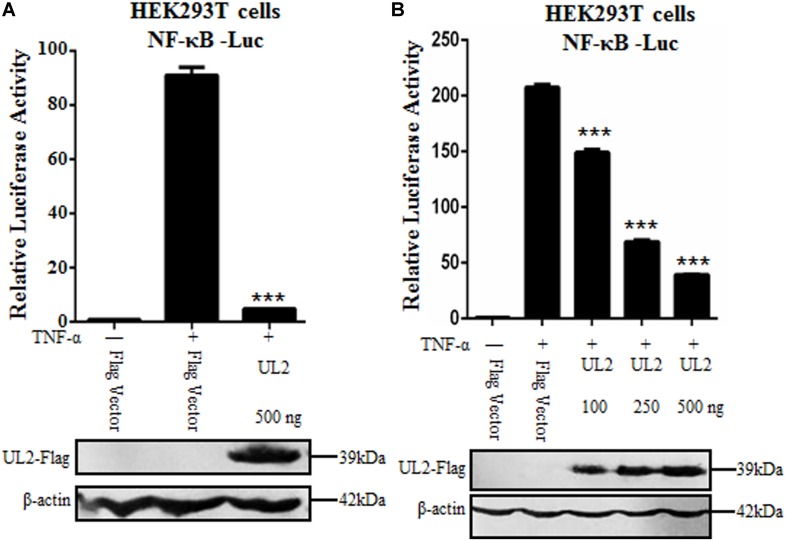
Inhibition of TNF-α–induced NF-κB activation by HSV-1 UL2. **(A)** HEK293T cells were transfected with promoter reporter plasmids NF-κB–Luc and pRL-TK, together with 500 ng of Flag empty vector or pUL2-Flag plasmid. Twenty-four hours posttransfection, cells were treated with or without 10 ng/mL of the recombinant human TNF-α and incubated for an additional 6 h, followed by cell lysed. Nuclear factor κB–driven luciferase activity was detected by DLR, as described in section “Materials and Methods.” **(B)** was carried out as **(A)**; except that for an increase indicated amounts (100, 250, and 500 ng) of UL2-Flag expression plasmid were used. Cell lysates were divided into two aliquots; one aliquot was used for DLR detection, and the other was used for WB analysis to detect the protein expression of transfected plasmid. The expression of UL2 was analyzed by WB with anti-Flag mAb, and β-actin was used to verify equal loading of protein in each lane. Dual-luciferase reporter data were normalized for transfection efficiency through measuring firefly luciferase activity and Renilla luciferase activity, and values were shown as the ratio between the firefly and Renilla luciferase. Data were expressed as means ± SD from three independent experiments. ***P < 0.001.

Nuclear factor κB plays a very important role in foreign virus infection, which can be activated by various stimuli. As a proinflammatory cytokine, TNF-α can be rapidly recognized by TNFR when stimulus signal acts on cells and then induces the activation of canonical NF-κB pathway (35). Here, we attempted to study whether HSV-1 UL2 protein can modulate NF-κB activity. Flag-tagged UL2 expression plasmid or Flag vector was cotransfected with reporter genes NF-κB–Luc and pRL-TK into HEK293T cells. Twenty-four hours posttransfection, cells were treated with TNF-α, and DLR assays were performed. As shown in Figure 1, TNF-α treatment resulted in strong induction of NF-κB promoter activity, but this activity was significantly down-regulated by ectopic expression of UL2 (Figure 1A). Moreover, when the concentration of UL2 was increased, UL2 down-regulated NF-κB promoter activity in a dose-dependent manner (Figure 1B). These data suggested that UL2 could inhibit TNF-α–induced NF-κB activation.

### UL2 Inhibits NF-κB–Regulated Cytokine Expression

**FIGURE 2:**
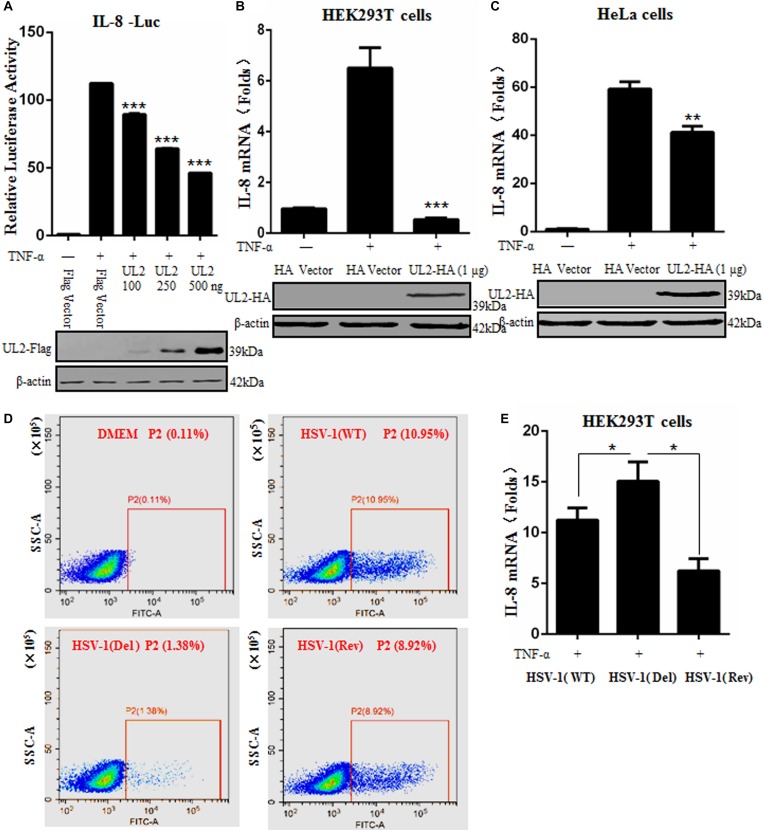
Herpes simplex virus 1 UL2 inhibits NF-κB–driven cytokine expression. **(A)** HEK293T cells were cotransfected with Flag vector or diverse concentrations (100, 250, and 500 ng) of UL2-Flag expression plasmid along with reporter plasmids pXP2-pIL-8-Luc and pRL-TK. Twenty-four hours posttransfection, cells were treated with TNF-α (10 ng/mL) for 6 h, and luciferase activity was measured as described in Figure 1. **(B)** HEK293T cells were transfected with 1 μg of HA control vector or UL2-HA expression plasmid; 24 h posttransfection, cells were treated with TNF-α (10 ng/mL) for 6 h, and then RT-qPCR analysis was performed to analyze the relative expression level of IL-8 mRNA. Glyceraldehyde-3-phosphate dehydrogenase was used as the housekeeping gene. **(C)** was carried out as **(B)**, except that HeLa cells were used for transfection. The expression of UL2 was analyzed by WB using anti-Flag mAb or anti-HA mAb, and β-actin was used to verify equal loading of protein in each lane. **(D)** HEK293T cells were mock-infected or infected with WT, UL2 Del, or UL2 Rev HSV-1 BAC GFP Luc virus at an MOI of 1 for 16 h. Flow cytometry analysis was then carried out to detect the GFP fluorescence. **(E)** HEK293T cells were infected with WT, UL2 Del, or UL2 Rev HSV-1 BAC GFP Luc virus at an MOI of 1. Sixteen hours postinfection, cells were treated with TNF-α (10 ng/mL) for 6 h. Then, RT-qPCR analysis was performed to detect the relative expression level of IL-8 mRNA. Data were expressed as means ± SD from three independent experiments. *P < 0.05, **P < 0.01, and ***P < 0.001.

After TNF-α stimulation, activated NF-κB can induce the expression of various proinflammatory cytokines, such as IL-1, IL-6, and IL-8 (19). To further study whether UL2 also can inhibit downstream NF-κB–regulated inflammatory cytokine expression, DLR assays were performed to observe the IL-8 promoter activity in the presence of UL2. Flag-tagged UL2 expression plasmid or Flag vector was cotransfected with reporter genes pXP2-pIL-8-Luc and pRL-TK into HEK293T cells. Cells were then treated or mock-treated with TNF-α, and then IL-8 luciferase activity was tested. As a result, IL-8 promoter luciferase activity was effectively activated by TNF-α, but this activation was significantly inhibited by UL2 in a dose-dependent fashion (Figure 2A).

To further investigate the inhibitory role of UL2 in TNF-α–regulated IL-8 production, IL-8 mRNA accumulation in HEK293T cells was measured by RT-qPCR. As shown in Figure 2B, IL-8 mRNA was successfully induced by TNF-α stimulation. However, its mRNA accumulation was strongly negatively regulated by UL2. Moreover, to confirm that the inhibition of cytokine expression by UL2 is not cell-specific, the IL-8 mRNA accumulation was also detected in HeLa cells, and result showed that TNF-α–induced IL-8 mRNA also could be reduced by UL2 (Figure 2C).

In order to validate the physiological function of UL2 in inhibiting proinflammatory cytokine expression during HSV-1 infection, we first explored whether UL2 affects the replication of HSV-1. HEK293T cells were mock-infected or infected with WT, UL2 Del, or UL2 Rev HSV-1 BAC GFP Luc for 16 h, and then flow cytometry analysis was performed. As results, low fluorescence (0.11%) could be detected in the uninfected cells, whereas 10.95% of cells with fluorescence could be detected when the cells were infected with WT HSV-1, but only 1.38% was detected when the cells were infected with UL2 Del HSV-1. In addition, this value rose to 8.92% when cells were infected with UL2 Rev HSV-1 (Figure 2D). Accordingly, these results indicated that UL2 indeed could affect the proliferation of HSV-1, which is consistent with our recent study (41).

Subsequently, HEK293T cells infected with WT, UL2 Del, or UL2 Rev HSV-1 BAC GFP Luc for 16 h were treated with TNF-α, and then the relative RT-qPCR analysis was performed according to previous studies (40, 47, 56–59), to test the IL-8 mRNA level. As shown in Figure 2E, WT HSV-1 infection induced a trace amount of IL-8 mRNA after TNF-α stimulation, but the UL2 Del HSV-1 induced significantly higher level of IL-8 mRNA than WT HSV-1 did, and the accumulation of IL-8 mRNA induced by UL2 Rev HSV-1 was similar to that of the WT HSV-1. Taken together, these results demonstrated the significant inhibitory effect of UL2 on NF-κB–regulated IL-8 inflammatory cytokine expression.

### UL2 Targets at or Downstream of p65 Level to Inhibit NF-κB Pathway

**FIGURE 3:**
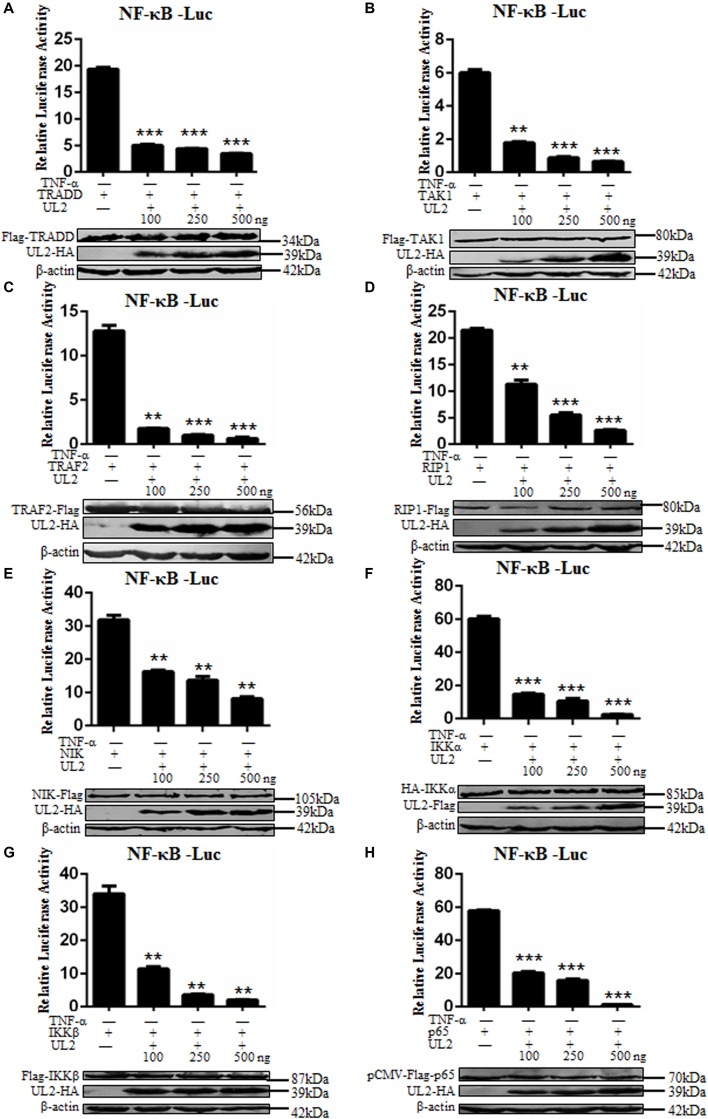
Herpes simplex virus 1 UL2 targets at or downstream of the p65 level to suppress NF-κB pathway. HEK293T cells were cotransfected with reporter plasmids NF-κB–Luc, pRL-TK, and 100 ng of TRADD **(A)**, TAK1 **(B)**, TRAF2 **(C)**, RIP1 **(D)**, NIK **(E)**, IKKα **(F)**, IKKβ **(G)**, or p65 **(H)** expression plasmid along with the indicated amounts (100, 250, and 500 ng) of UL2-Flag or UL2-HA expression plasmid, and then luciferase activity was analyzed as described in Figure 1. Cell lysates were analyzed by WB with corresponding tag-specific Abs to detect the expression of related plasmids, and β-actin was used to verify equal loading of protein in each lane. Data were expressed as means ± SD from three independent experiments. **P < 0.01 and ***P < 0.001.

Tumor necrosis factor α activates classical NF-κB pathway through the role of components including TRADD, TRAF2, RIP1, TAK1, IKKα, IKKβ, and p65, and overexpression of these signaling components can efficiently activate NF-κB pathway without TNF-α stimulation (40, 56, 60, 61). In order to probe at what level UL2 can inhibit the NF-κB activation, NF-κB–Luc, pRL-TK, increasing indicated amounts of Flag-tagged or HA-tagged UL2 expression plasmid, and the canonical NF-κB pathway component expression plasmid were cotransfected into HEK293T cells. Nuclear factor κB–inducing kinase (NIK), an important component in non-classical NF-κB pathway, was also cotransfected into cells, and the relative quantitative analysis was performed according to previous studies (40, 47, 56–59). As a result, NF-κB promoter was effectively activated by all of the mentioned NF-κB pathway components, which were blocked by UL2 in a dose-dependent manner (Figures 3A–H). Thus, these data proved that UL2 might target at or downstream of p65 to inhibit the NF-κB pathway.

### UL2 Interacts With Endogenous p65 and p50

**FIGURE 4:**
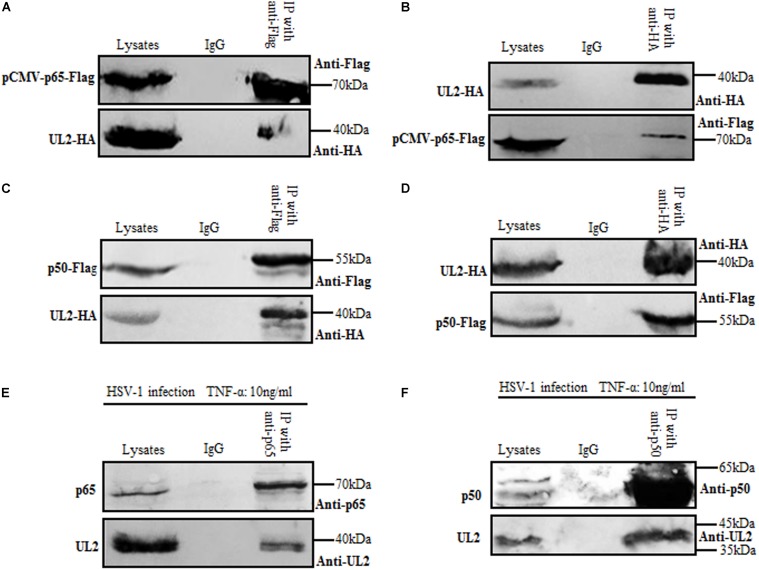
Herpes simplex virus 1 UL2 interacts with endogenous p65 and p50. **(A,C)** HEK293T cells were cotransfected with pCMV-p65-Flag and pUL2-HA **(A)** or p50-Flag and pUL2-HA **(C)** expression plasmids. Twenty-four hours posttransfection, cells were harvested and lysed, and the samples were then subjected to Co-IP assays using anti-Flag mAb or non-specific mouse IgG. Western blots were probed with the indicated Abs. **(B,D)** HEK293T cells were cotransfected with plasmids as described for **(A,C)**, respectively. Protein lysates were Co-IPed using anti-HA mAb, and WBs were analyzed with the indicated Abs. **(E,F)** HEK293T cells infected with WT HSV-1 at an MOI of 1 for 24 h were treated with 10 ng/mL of the TNF-α for an additional 6 h. Cells were then lysed, and the extracts were subjected to Co-IP using anti-p65 pAb **(E)**, anti-p50 pAb **(F)**, or control IgG. Samples were analyzed by WBs with the indicated Abs.

In order to gain insights into the inhibition mechanism of NF-κB activation by UL2, the cellular interaction between UL2 and NF-κB subunit p65 or p50 was explored by Co-IP. HEK293T cells were overexpressed with pUL2-HA and pCMV-p65-Flag, which can activate the NF-κB signaling. Then, reciprocal Co-IP analyses were performed with either anti-Flag (Figure 4A) or anti-HA (Figure 4B) mAb, and results demonstrated that UL2 could be mutually Co-IPed with p65. Besides, similar results between UL2 and p50 were obtained when HEK293T cells were cotransfected with pUL2-HA and p50-Flag expression plasmids, of which the reciprocal Co-IP analyses were also performed with anti-Flag (Figure 4C) or anti-HA (Figure 4D) mAb, suggesting UL2 could interact with p65 and p50.

To further investigate the interaction between UL2 and endogenous p65 or p50 during HSV-1 infection, HEK293T cells infected with HSV-1 for 24 h were treated with TNF-α for an additional 6 h to activate the NF-κB signaling, and then Co-IP assays were performed with either anti-p65 (Figure 4E) or anti-p50 (Figure 4F) pAb. As shown in Figure 4, UL2 could be efficiently Co-IPed with p65 or p50 in the presence of TNF-α, using anti-p65 (Figure 4E) or anti-p50 (Figure 4F) pAb, but not the control antibody IgG. Taken together, these data indicated that UL2 could interact with endogenous p65 and p50 during HSV-1 infection.

### The Region of Amino Acids 9 to 17 Is Responsible for UL2 Inhibition Through Interacting With p65 and p50

**FIGURE 5:**
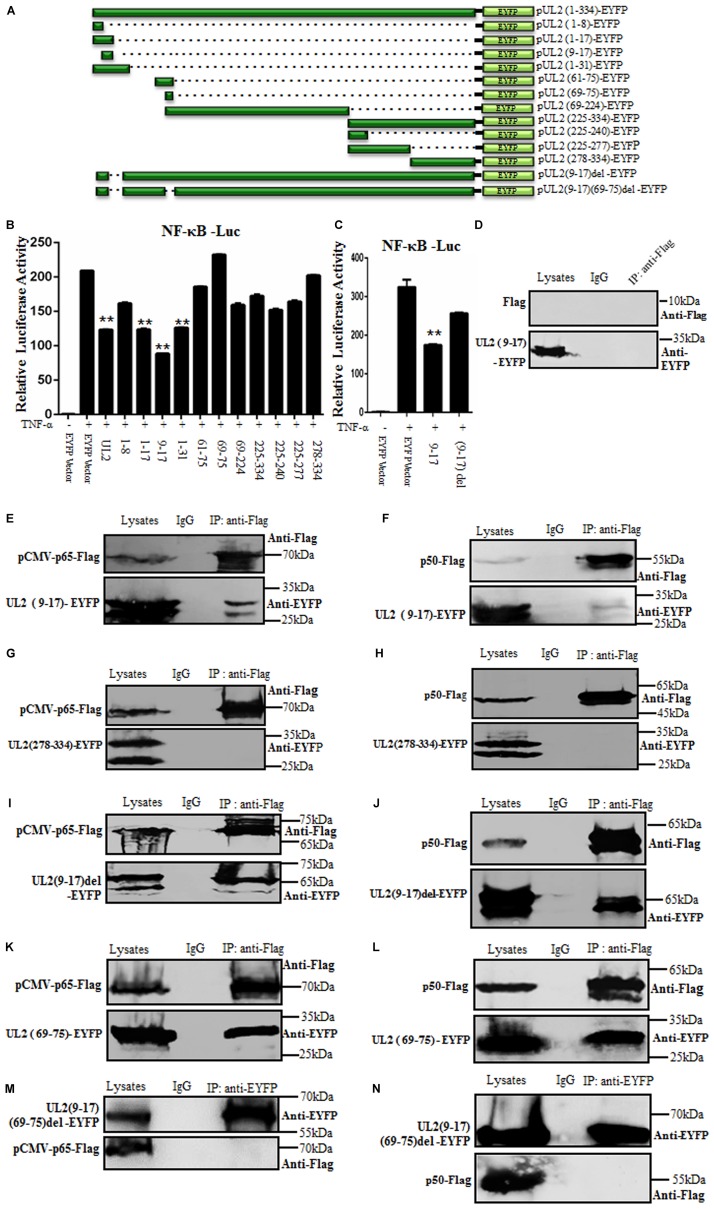
The region of aa9-17 is responsible for HSV-1 UL2 inhibition of NF-κB activity through interacting with p65 and p50. **(A)** Schematic representations of WT and truncated mutants of UL2 constructed in our laboratory. **(B,C)** HEK293T cells were cotransfected with NF-κB–Luc and pRL-TK reporter plasmids, along with 500 ng of EYFP empty vector or plasmid encoding EYFP-fused WT or truncated mutants of UL2. Twenty-four hours posttransfection, cells were treated with or without TNF-α (10 ng/mL) for 6 h, and luciferase activity was analyzed, as described in Figure 1. Data were expressed as means ± SD from three independent experiments. **(D–N)** HEK293T cells were cotransfected with pUL2(9-17)-EYFP and Flag vector **(D)**, pUL2(9-17)-EYFP and pCMV-p65-Flag **(E)**, pUL2(9-17)-EYFP and p50-Flag **(F)**, pUL2(278-334)-EYFP and pCMV-p65-Flag **(G)**, pUL2(278-334)-EYFP and p50-Flag **(H)**, pUL2(9-17)del-EYFP and pCMV-p65-Flag **(I)**, pUL2(9-17)del-EYFP and p50-Flag **(J)**, pUL2(69-75)-EYFP and pCMV-p65-Flag **(K)**, pUL2(69-75)-EYFP and p50-Flag **(L)**, pUL2(9-17)(69-75)del-EYFP and pCMV-p65-Flag **(M)**, or pUL2(9-17)(69-75)del-EYFP and p50-Flag **(N)** expression plasmids. Twenty-four hours posttransfection, cells were harvested and lysed, and the samples were then subjected to Co-IP assays using anti-Flag mAb, anti-EYFP or non-specific mouse IgG. Western blots were probed with the indicated Abs. **P < 0.01.

To clarify the region of UL2 required for the inhibition of NF-κB activation, a series of truncated mutants of UL2 fused to the N-terminus of EYFP were constructed (Figure 5A). Then, WT or UL2 mutants or EYFP vector expression plasmid was transfected into HEK293T cells to detect their ability to inhibit TNF-α–induced NF-κB activation by DLR assays. Compared to WT UL2, only aa1-17, aa9-17, and aa1-31 truncated mutants could significantly inhibit TNF-α–induced NF-κB promoter activity (Figure 5B), but aa9-17 deletion [UL2(9-17)del] reduced the UL2-mediated inhibition effect of NF-κB activation (Figure 5C), suggesting the minimum fragment aa9-17 might be important for UL2 inhibitory activity.

To further support this hypothesis, the interaction between aa9-17 of UL2 and p65 or p50 was analyzed. UL2(9-17)-EYFP or UL2(278-334)-EYFP (negative control, which does not inhibit NF-κB activity) was cotransfected with either pCMV-p65-Flag, p50-Flag, or Flag vector expression plasmid into HEK293T cells, and then Co-IP/WB experiments were performed. As results, UL2(9-17)-EYFP (possesses two bands, Figures 5E,F), but not UL2(278-334)-EYFP (contains two bands) (Figures 5G,H), was efficiently Co-IPed by p65 (Figure 5E) and p50 (Figure 5F) with anti-Flag mAb, whereas no such protein was immunoprecipitated with Flag vector control (Figure 5D) or control mouse IgG, which was consistent with the DLR results (Figure 5B). To further determine whether aa9-17 is the only domain that can bind to p65 and p50, the interaction between aa9-17 deletion of UL2 and p65 or p50 was tested, and results showed that UL2(9-17)del-EYFP (includes two bands) still could interact with p65 and p50 (Figures 5I,J), suggesting there are other areas that can interplay with p65 and p50. Therefore, UL2(69-75), which also does not inhibit NF-κB promoter activity (Figure 5B), was cotransfected for Co-IP with pCMV-p65-Flag or p50-Flag. As shown in Figure 5, UL2(69-75) could associate with p65 and p50 (Figures 5K,L). To further confirm this result, a double-deletion mutant UL2 (9–17) (69–75) del-EYFP expression plasmid was constructed and used to analyze its interaction with p65 or p50, and results showed that UL2(9–17) (69–75) del-EYFP could not interact with either p65 or p50 (Figures 5M,N). Consequently, these evidences demonstrated that aa69-75 can assist the interaction between UL2 and p65 or p50, but aa9-17 is responsible for the inhibition of NF-κB activity through interacting with NF-κB subunits p65 and p50.

### IPT Domain of p65 Interacts With UL2

**FIGURE 6:**
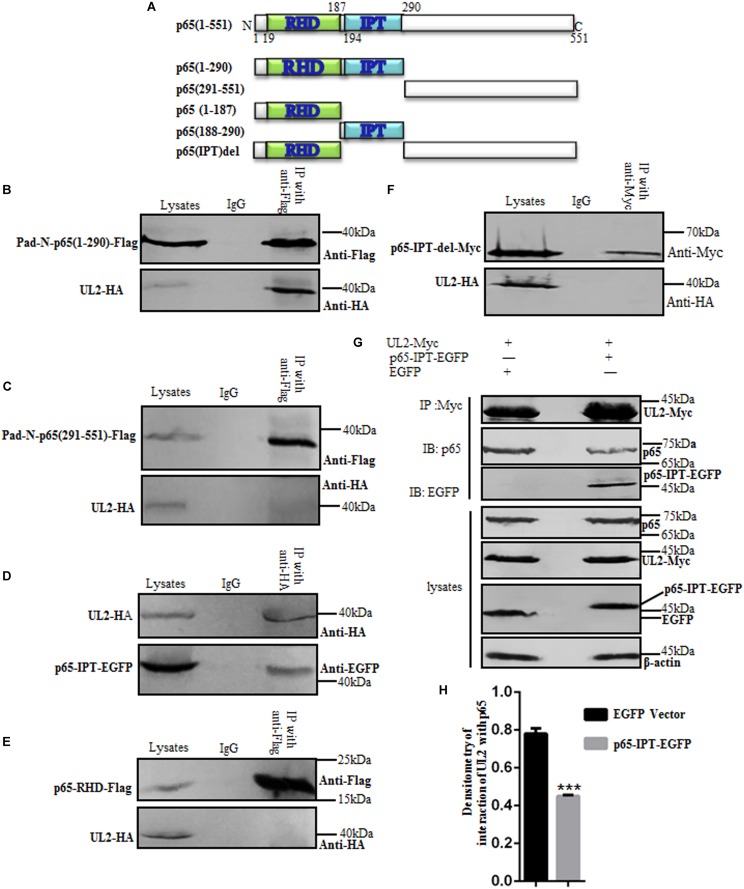
p65-IPT is associated with HSV-1 UL2. **(A)** Structural analysis diagrams of p65. RHD denotes Rel homology domain, and IPT denotes immunoglobulin-like plexin transcription factor. **(B–F**) HEK293T cells cotransfected with pUL2-HA and p65 truncated construct, including Pad-N-p65(1-290)-Flag **(B)**, Pad-N-p65(291-551)-Flag **(C)**, p65-IPT-EGFP **(D)**, p65-RHD-Flag **(E)**, or p65-IPT-del-Myc **(F)**, were analyzed by Co-IP assays. **(B,C,E)** were Co-IPed with anti-Flag mAb; **(D,F)** were Co-IPed with anti-HA **(D)** and anti-Myc **(F)** mAbs, respectively. Western blots were probed with the indicated Abs. **(G)** HEK293T cells were cotransfected with expression plasmids UL2-Myc and p65-IPT-EGFP or EGFP vector. Twenty-four hours posttransfection, cell lysates were harvested and analyzed by Co-IP assays with anti-Myc mAb, and WBs were performed with the indicated Abs. Expression level of β-actin was served as loading control. **(H)** Densitometry of the UL2 and endogenous p65 protein interaction bands was normalized to β-actin. Data were expressed as means ± SD from three independent experiments. ***P < 0.001.

It is reported that the N-terminal Rel homology domain (RHD) of p65 (p65-RHD) is the key domain for the dimerization, nuclear import, and DNA binding of NF-κB to promote gene transcription (62), and the IPT domain of p65 (p65-IPT) is also an important functional region for the dimerization of p65/p50 (45). To investigate the critical region of p65 for its interaction with UL2, aa1–290 or aa291–551 of p65 expression plasmid Pad-N-p65(1–290)-Flag or Pad-N-p65(291–551)-Flag was first cotransfected with pUL2-HA into HEK293T cells, and then Co-IP/WB experiments were performed. As shown in Figure 6, aa1–290 of p65 (Figure 6B), but not aa291–551 (Figure 6C), could associate with UL2. Because aa1-290 consists of RHD and IPT domains, the interaction between UL2-HA and p65-RHD-Flag or p65-IPT-EGFP expression plasmid was examined by Co-IP/WB experiments, as described above. As a result, p65-IPT (Figure 6D), but not p65-RHD (Figure 6E), bound to UL2.

To further determine the significance of IPT for the interaction between p65 and UL2, IPT deletion of p65 was constructed (p65-IPT-del-Myc) (Figure 6A) and cotransfected with UL2-HA into HEK293T cells for Co-IP assays. Result showed that UL2 could not interact with p65-IPT-del (Figure 6F); we therefore presumed that whether p65-IPT could act as a dominant negative to inhibit the interaction between UL2 and p65. To verify this hypothesis, p65-IPT-EGFP or EGFP vector cotransfected with pUL2-Myc expression plasmid into HEK293T cells for 24 h was harvested and analyzed for the interaction between UL2 and endogenous p65 by Co-IP assays using anti-Myc mAb. As shown in Figure 6, UL2 could interact with endogenous p65, but this interaction was significantly inhibited in the presence of p65-IPT (Figures 6G,H), indicating that IPT was essential for the interaction between p65 and UL2.

### UL2 Does Not Affect p65/p50 Dimerization

**FIGURE 7:**
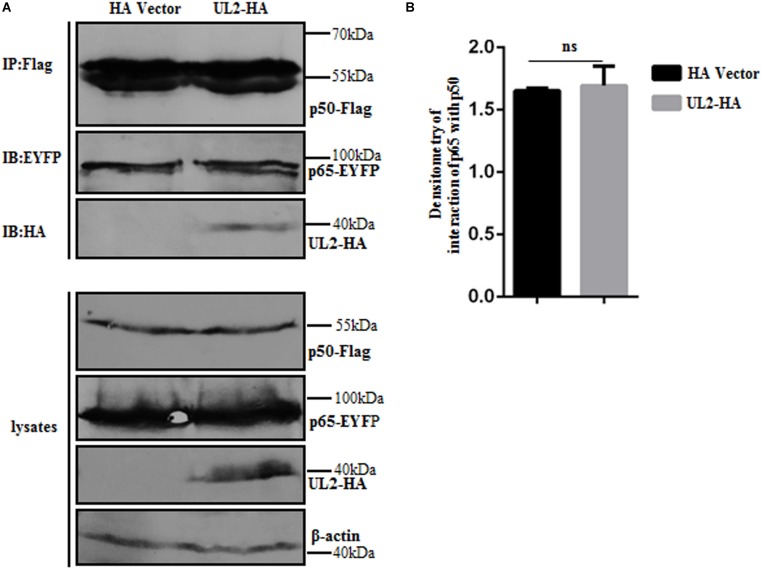
HSV-1 UL2 does not affect the dimerization of p65/p50. **(A)** HEK293T cells cotransfected with expression plasmids p65-EYFP, p50-Flag, and UL2-HA or HA vector construct for 24 h were harvested and analyzed by Co-IP assays using anti-Flag mAb, and WBs were performed using the indicated Abs. Expression level of β-actin was served as loading control. **(B)** Densitometry of the p65 and p50 interaction bands was normalized to β-actin. Data were expressed as means ± SD from three independent experiments. ns, not significant.

Because IPT is important for the formation of p65/p50 dimerization (45), we speculated whether the interaction between UL2 and p65-IPT could competitively inhibit the interaction between p65 and p50 and therefore affect the formation of heterodimer p65/p50. In order to verify this hypothesis, expression plasmids p65-EYFP and p50-Flag (overexpression can activate the NF-κB signaling) were cotransfected with pUL2-HA or HA vector into HEK293T cells, and then Co-IP, which is an accepted technique to detect protein–protein dimerization (such as p65/p50) (44, 63, 64), was carried out with anti-Flag mAb. As shown in Figure 7, there was no obvious difference in the interaction bands between p65 and p50 in the presence of HA vector or UL2, suggesting that UL2 did not influence the p65/p50 dimerization.

### UL2 Does Not Block TNF-α–Induced p65 and p50 Nuclear Translocation

**FIGURE 8:**
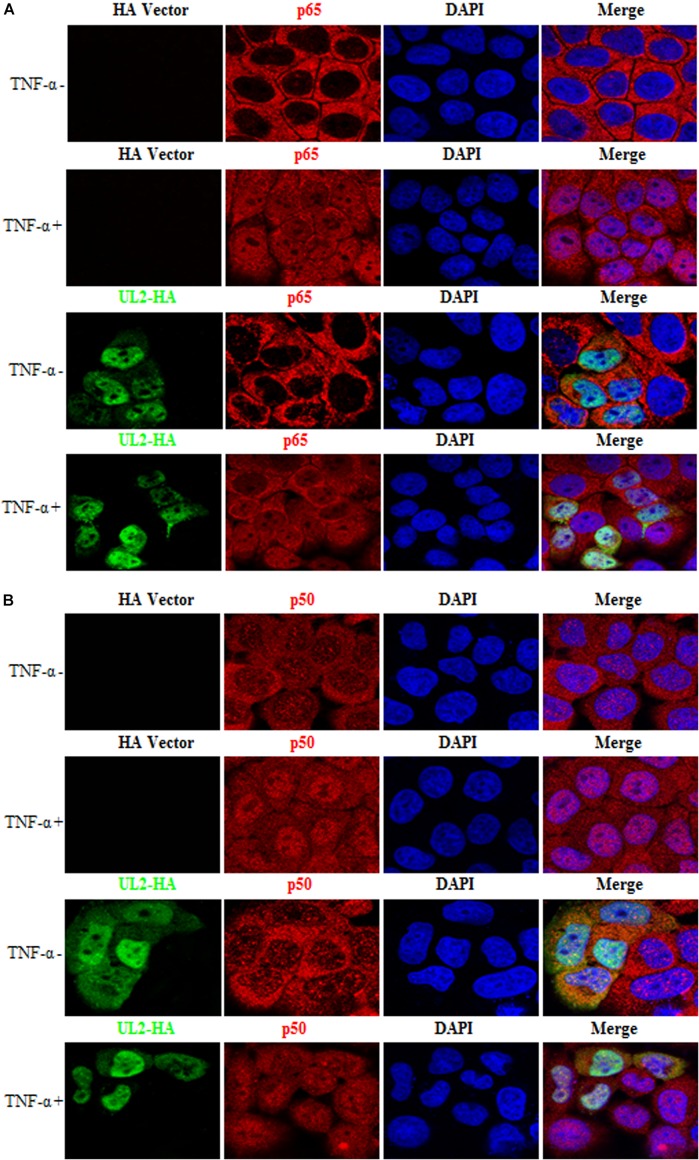
HSV-1 UL2 does not block the TNF-α–induced nuclear translocation of p65 or p50. HeLa cells were transfected with HA vector or UL2-HA expression plasmid. Twenty-four hours posttransfection, cells were treated with TNF-α (10 ng/mL) or mock-treated for 30 min. Then, cells were stained with anti-HA mAb and anti-p65 pAb **(A)** or anti-p50 pAb **(B)**. Fluorescein isothiocyanate–conjugated donkey anti–mouse IgG (green) and Cy5-conjugated goat anti–rabbit IgG (red) were used as the secondary Abs. Cell nuclei were stained with DAPI (blue). All of the transfected cells were analyzed by a confocal microscope (Axio-Imager-LSM-800; Zeiss), and the photomicrographs were taken at a magnification of 400×. Each image represented a vast majority of the cells with similar subcellular distribution. Statistical analysis of the subcellular localization of p65 or p50 in the absence or presence of UL2 is shown in Table 2.

After TNF-α binds to TNFR, IκBα is phosphorylated by IKKs, followed by its ubiquitination and degradation, and then the p65/p50 dimer was released and translocated into nucleus (30, 35, 36), which is important for the NF-κB activation. Here, HeLa cells, which are widely employed in different studies for indirect immunofluorescence assay as its nucleus, are obviously larger than cytoplasm (40, 56, 60, 61, 65–74), were used to test whether UL2 could block the nuclear translocations of p65 and p50. As shown in Figure 8 and statistical analysis of the subcellular localization in Table 2 that is widely applied in many studies (61, 67, 68, 70, 71), p65 and p50 were localized exclusively to the cytoplasm in the mock-stimulated HeLa cells, which were then translocated into the nuclei after TNF-α treatment. However, ectopic expression of UL2 could not restrict TNF-α–mediated nuclear accumulation of p65 or p50. These results revealed that the binding of UL2 to p65-IPT was insufficient to prohibit the nuclear translocation of NF-κB.

**TABLE 2:** Subcellular localization of NF-κB in the presence of HSV-1 UL2.

Subunit of NF-κB	Cells transfected with vector or UL2 expression plasmid	Cells treated with TNF-α	Total number of cells transfected with vector or UL2 expression plasmid	Subcellular localization pattern of NF-κB subunit in cells transfected with vector or UL2 expression plasmid	Subcellular localization change of NF-κB subunit in cells transfected with vector or UL2 expression plasmid	Number of subcellular localization change of NF-κB subunit in cells transfected with vector or UL2 expression plasmid	Percentage of subcellular localization change of NF-κB subunit in cells transfected with vector or UL2 expression plasmid
p65	HA vector	−	50	Pan-cytoplasmic	No	0	0
p65	HA vector	+	50	Pan-cellular	No	2	4
p65	UL2-HA	−	50	Pan-cytoplasmic	No	0	0
p65	UL2-HA	+	50	Pan-cellular	No	1	2
p50	HA vector	−	50	Pan-cytoplasmic	No	0	0
p50	HA vector	+	50	Pan-cellular	No	2	4
p50	UL2-HA	−	50	Pan-cytoplasmic	No	0	0
p50	UL2-HA	+	50	Pan-cellular	No	2	4

*HeLa cells were transfected with HA vector or UL2-HA expression plasmid. 24 h post-transfection, cells were treated with TNF-α (10 ng/ml) or mock-treated for 30 min. Then, cells were examined for the subcellular localization pattern of p65 or p50 by IFA.*

### p65 Phosphorylation Is Suppressed by UL2

**FIGURE 9:**
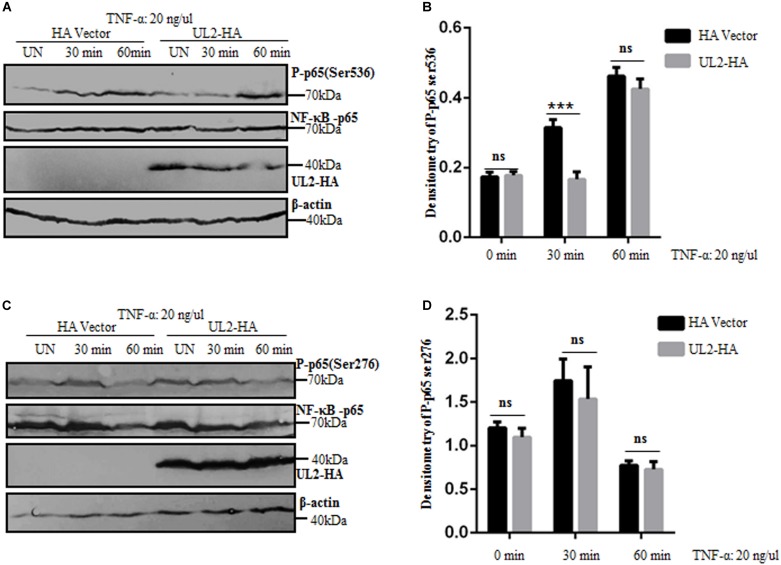
p65 phosphorylation at Ser536 is suppressed by HSV-1 UL2. **(A,C)** HEK293T cells transfected with either HA empty vector or UL2-HA expression plasmid were stimulated with TNF-α (20 ng/mL) for the indicated times (0, 30, and 60 min) according to previous studies (59, 77), and then equal amounts of cell lysates were analyzed by WBs with phospho-NF-κB–p65 (Ser536) Ab **(A)**, phospho-NF-κB–p65 (Ser276) Ab **(C)** (top panel), or anti-p65 pAb (second panel). Protein levels of UL2 (third panel) and β-actin (bottom panel) in the same cell lysates were also determined. **(B,D)** Densitometry of phospho-NF-κB–p65 Ser536 **(B)** and Ser276 bands **(D)** from **(A,C)**, respectively, were normalized to loading control β-actin. Data were expressed as means ± SD from three independent experiments. ns, not significant and ***P < 0.001.

Regulation of NF-κB activity is crucial for the selection and transcriptional activity of NF-κB target genes. Various posttranslational modifications influence the binding affinity of NF-κB to DNA and its interactions with coactivators and coreceptors (75, 76). p65 phosphorylation is an important modification of NF-κB to regulatory its activity. It has been reported that p65 phosphorylation occurs at multiple sites, and the most vital phosphorylations are Ser536 and Ser276 (76). The aforementioned results demonstrated that UL2 targeted at the p65 level and interacted with endogenous p65 and p50 to inhibit NF-κB pathway. To further clarify the inhibition mechanism of NF-κB activation by UL2, the effect of UL2 on p65 phosphorylation was examined. HEK293T cells were transfected with pUL2-HA or HA vector; 24 h posttransfection, cells were treated with TNF-α for 0, 30, and 60 min according to previous studies (59, 77), and then the phosphorylations of p65 (Ser536) and p65 (Ser276) were detected by WB, which is a usual technique to detect p65 phosphorylation (76, 78, 79). As shown in Figure 9, the total amount of p65 expression was the same in each sample, whereas the phosphorylation of p65 (Ser536) was significantly inhibited by UL2, and the inhibitory effect was approximately 3 times when cells were TNF-α–treated for 30 min. However, the inhibitory effect was not noticeable when cells were treated with TNF-α for 60 min (Figures 9A,B). Besides, UL2 had no effect on the phosphorylation of p65 (Ser276) when cells were treated with TNF-α for 30 or 60 min (Figures 9C,D). Overall, these results indicated that UL2 could suppress the p65 phosphorylation at Ser536 to inhibit NF-κB activation.

## Discussion

The innate immune system, an evolutionarily ancient form of host defense, is the first line against pathogens (80, 81). It recognizes specific components of microorganisms, including toxoid lipopolysaccharide, bacterial flagellin, and viral nucleic acid and then initiates signaling cascades and eventually induces the activation of various transcription factors or expression of multiple inflammatory factors and antiviral proteins, which served as a host strategy to block pathogen invasion and spread within cells (82). It is well known that NF-κB is a critical regulator of the host innate immune system. Nuclear factor κB binds to downstream gene enhancer or promoter sequences, which ultimately mediates gene transcription and expression, leading to the up-regulation of hundreds of different chemokines and cytokines and expression of a variety of antiviral proteins (83).

In order to survive in the host, viruses have evolved and developed different mechanisms to evade host’s antiviral response. As we all know, NF-κB provides an attractive target to different viruses for modulating host TNF-mediated events (30). Therefore, NF-κB signaling pathway deserves to be the victim challenged by these viruses. In the early infection, 073 protein of orf virus is reported to inhibit TNF-α–induced NF-κB activation through the interaction with IKK regulatory subunit NEMO to decrease the phosphorylation of IKKα, IKKβ, and IκBα, which finally blocks the nuclear translocation of p65 (84). The primate simian immunodeficiency virus–encoded Vpr protein can target IKK complex and stabilize IκBα to inhibit p65 phosphorylation, which subsequently block the NF-κB–dependent immune activation (85). Herpes simplex virus 1 also can encode some proteins to affect the transcriptional activation of NF-κB. For example, UL24, UL42, US3, and ICP0 are shown to inhibit TNF-α–induced NF-κB activation by preventing the nuclear translocation of NF-κB (40, 56, 60, 61). In our study, we demonstrated that UL2 protein could interact with endogenous p65 and p50 to inhibit NF-κB activity by attenuating TNF-α–induced p65 phosphorylation at Ser536, which therefore could down-regulate the transcription of downstream IL-8. Currently, the specific mechanism of how p65 Ser536 phosphorylation induces downstream signal transmission is not clear. It is reported that phosphorylation at Ser536 in the C-terminal transactivation domain (TAD) of p65 leads to enhanced transactivation of NF-κB–dependent target genes (such as IL-8) transcription, through increased CBP/p300 binding and acetylation at K310 of p65 (77, 86–90).

Immunoglobulin-like plexin transcription factor is a key domain for p65 to form p65/p50 (45). Study shows that the 2C protein of enterovirus 71 can interact with p65-IPT and disrupt p65/p50 heterodimer to suppress NF-κB activation (44). However, UL2 could not affect the formation of p65/p50 dimerization in our study. Besides, p65/p50 nuclear translocation is essential for the transcription activity of NF-κB (56). Thus, we wondered if UL2 could hijack p65 and p50 in the cytoplasm to prevent NF-κB activation. Regrettably, UL2 could not inhibit TNF-α–induced nuclear translocation of p65 or p50, which may due to the reason that RHD of p65 or p50 is also crucial for the dimerization, DNA binding, and nuclear import of NF-κB (62), and UL2 only could interact with the IPT domain of p65.

Nuclear factor κB posttranslational modification is also critical for recruiting the transcriptional apparatus and stimulating target gene expression (91–93). Phosphorylation of NF-κB involves the release of NF-κB from IκB, nuclear transport, processing of precursors, stabilization of dimerization, and DNA binding (94). Study has shown that p65 phosphorylation at Ser536 is required for p65 transactivation, which also affects p65 binding to DNA and the recruitment of p300 (92). Furthermore, p65 phosphorylation at Ser276 can lead to a conformational change of p65, exposing the p65 C-terminal TAD, thereby to promote its transcriptional activity (93). Pollock and colleagues (95) demonstrate that the small hydrophobic protein of bovine respiratory syncytial virus can inhibit TNF-α–induced p65 phosphorylation at Ser536, which is responsible for blocking the NF-κB pathway. Moreover, orf virus 002 protein can inhibit p65 phosphorylation at Ser276 and block the acetylation of p65, which eventually reduce NF-κB activity (76). In the present study, we identified that UL2 inhibited the phosphorylation of p65 (Ser536) at 30 min after TNF-α treatment, and the inhibitory effect is approximately three times when compared to HA vector. However, the inhibition effect is not noticeable at 60 min. The most probable reason for this result is due to that TNF-α–induced p65 phosphorylation at Ser536 is activated after stimulation for 10 min and then gradually increased and peaked at 60 min. It starts to decrease after 2 h, and the expression level is restored to the same amount as no treatment when stimulation is extended to 4 h (77). At the beginning of TNF-α stimulation, UL2 inhibited p65 phosphorylation (Ser536) at 30 min, but when the p65 phosphorylation was achieved to the strongest at 60 min, accumulation of phosphorylated p65 reached the maximum, presenting that the UL2 inhibitory effect was not noticeable. In other words, UL2 inhibited p65 phosphorylation (Ser536) at an early stage of the p65 phosphorylation cycle. It is therefore reasonable that inhibition by UL2 follows the periodic oscillation of p65 phosphorylation (Ser536). Additionally, UL2 had no significant effect on the p65 phosphorylation at Ser276.

According to our DLR results, UL2 not only could inhibit the NF-κB activation through classical pathway, but also could effectively inhibit the NF-κB promoter activity activated by the non-canonical NF-κB signaling pathway components of IKKα and NIK, indicating UL2 might also be an antagonist of the non-canonical NF-κB signaling pathway.

The relationship between HSV-1 infection and innate immunity is rather complex. The coexistence of the virus and host must come to a state of balance. The same is true for the balance between HSV-1 and NF-κB immune response. After HSV-1 infection, certain proteins including UL31 (96), UL37 (97), glycoprotein B (gB), gH, gL (47, 98, 99), and gD (100) can transiently activate the classical NF-κB pathway and up-regulate the expression of some host genes to facilitate viral replication and propagation. It is proven that the host protein IκBα contributes to the effective replication of HSV-1, and IKKα and IKKβ also contribute to the accumulation of viral production in HSV-1–infected cells (101). However, NF-κB is not infinitely activated, the inflammatory cytokines induced by NF-κB in turn can inhibit viral infection. Thus, HSV-1 may transiently activate NF-κB to initiate its early infection and then escape the immune surveillance with its viral gene products, to balance between virus replication and host antiviral response. To date, UL2, UL24, UL42, US3, and ICP0 are demonstrated to possess the competence to suppress TNF-α–induced NF-κB activity (40, 56, 60, 61), which are considered equally important for the synergetic inhibition of NF-κB activity at various levels of NF-κB signaling pathway during diverse stages of HSV-1 life cycle.

## Conclusion

In conclusion, here we demonstrated that UL2 was an antagonistic protein for negatively regulating NF-κB signaling pathway by impairing the phosphorylation of p65 at Ser536 to interrupt the host’s antiviral response. Accordingly, UL2 may be an important regulator for the balance between HSV-1 infection and host NF-κB innate immunity.

## Data Availability Statement

The raw data supporting the conclusions of this article will be made available by the authors, without undue reservation, to any qualified researcher.

## Author Contributions

MC and ML designed the research. MC, ZL, XZ, ZX, YW, TL, YL, XO, YD, and YG performed the research. MC, ZL, and ML analyzed the data. TP consulted and advised on the research. MC, ZL, and ML wrote and reviewed the manuscript. All authors read and approved the final manuscript.

## Conflict of Interest

TP was employed by South China Vaccine Corporation Limited. The remaining authors declare that the research was conducted in the absence of any commercial or financial relationships that could be construed as a potential conflict of interest.
