# Supplementary material for: Herpes Simplex Virus 1 UL2 Inhibits the TNF-α–Mediated NF-κB Activity by Interacting With p65/p50
Source: Front Immunol. 2020 May 13;11:549. doi: 10.3389/fimmu.2020.00549 (PMC7237644; doi:10.3389/fimmu.2020.00549)

Original western blot results of Fig. 1

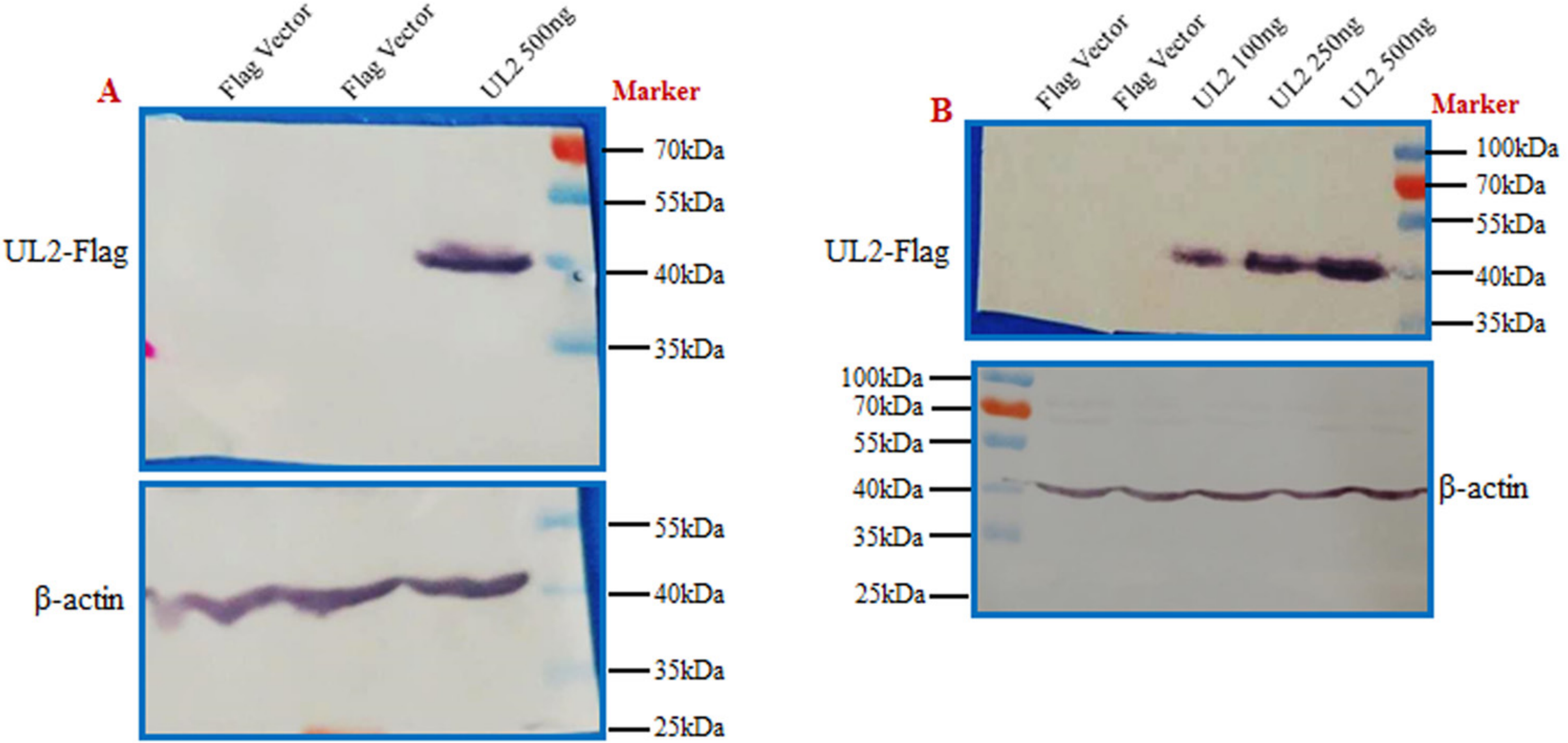

Original western blot results of Fig. 2

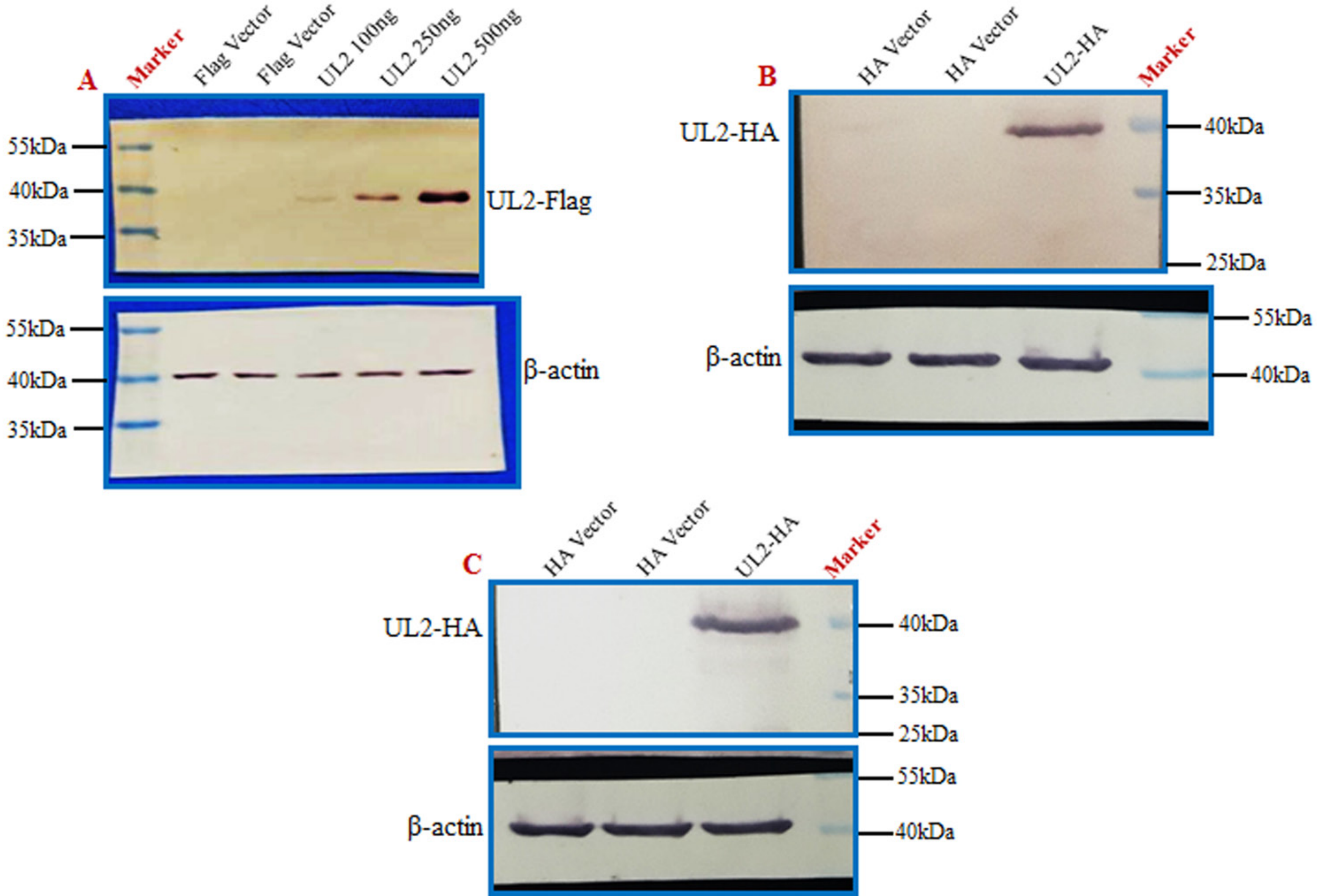

Original western blot results of Fig. 3

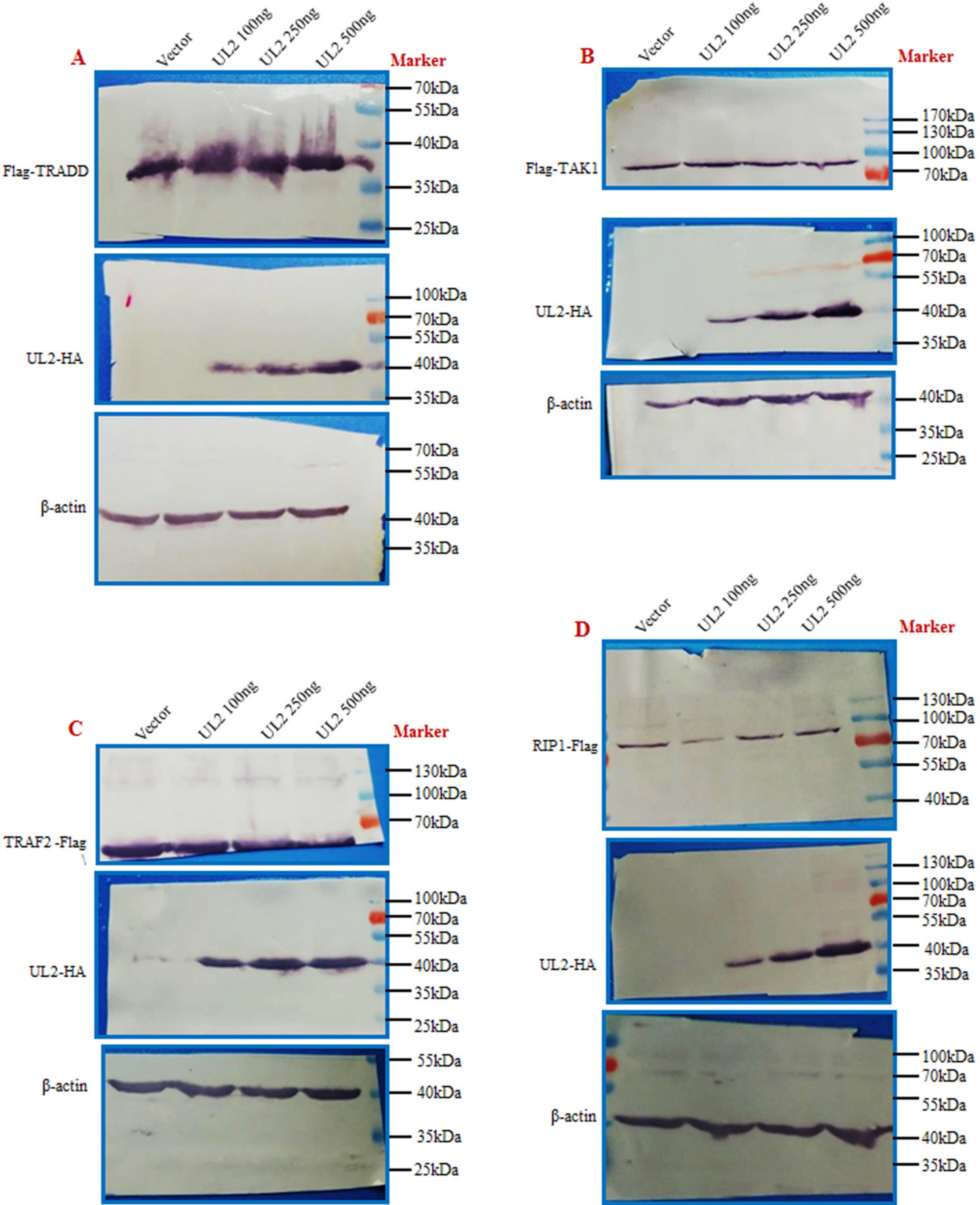

Original western blot results of Fig. 3

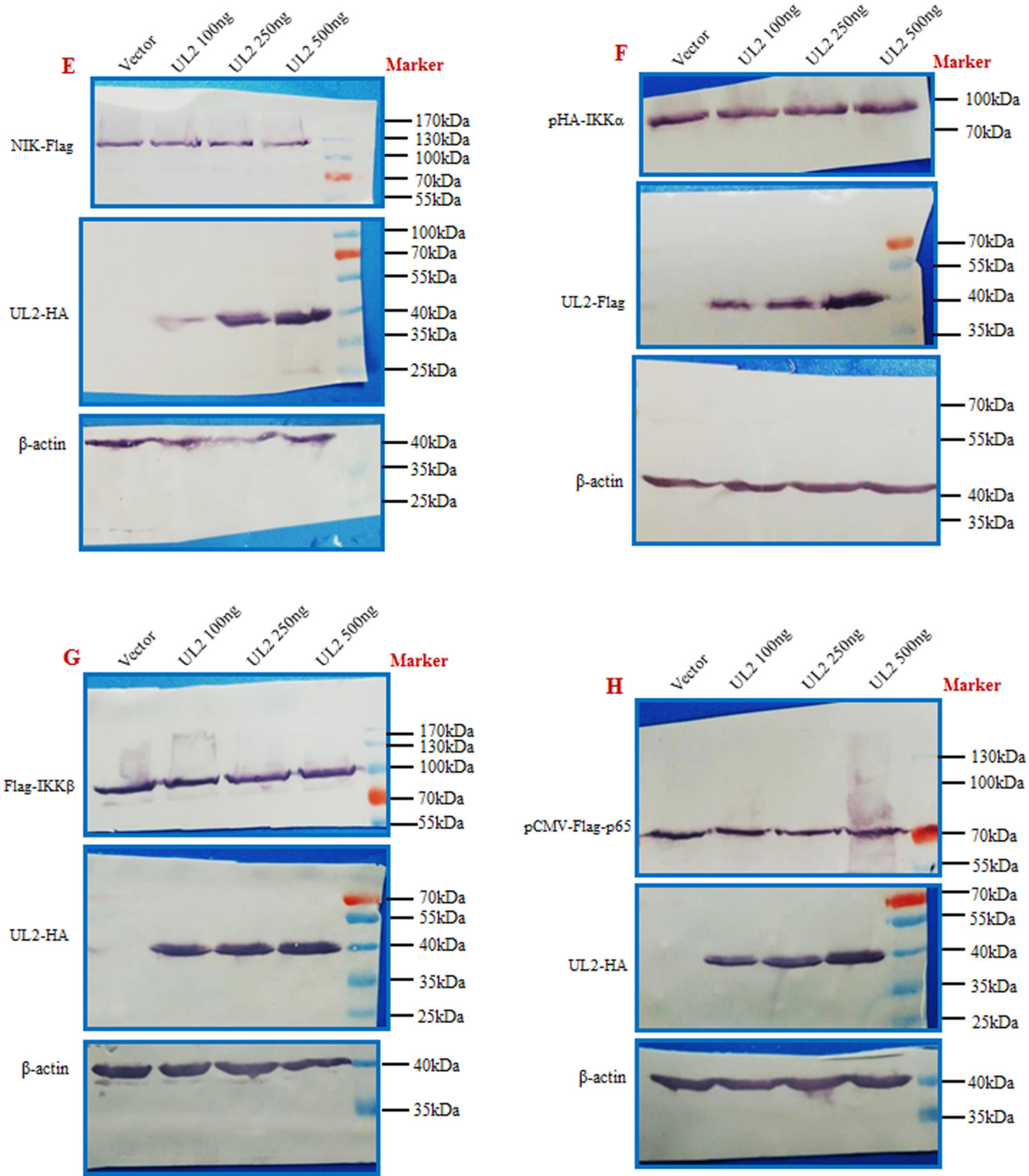

Original western blot results of Fig. 4

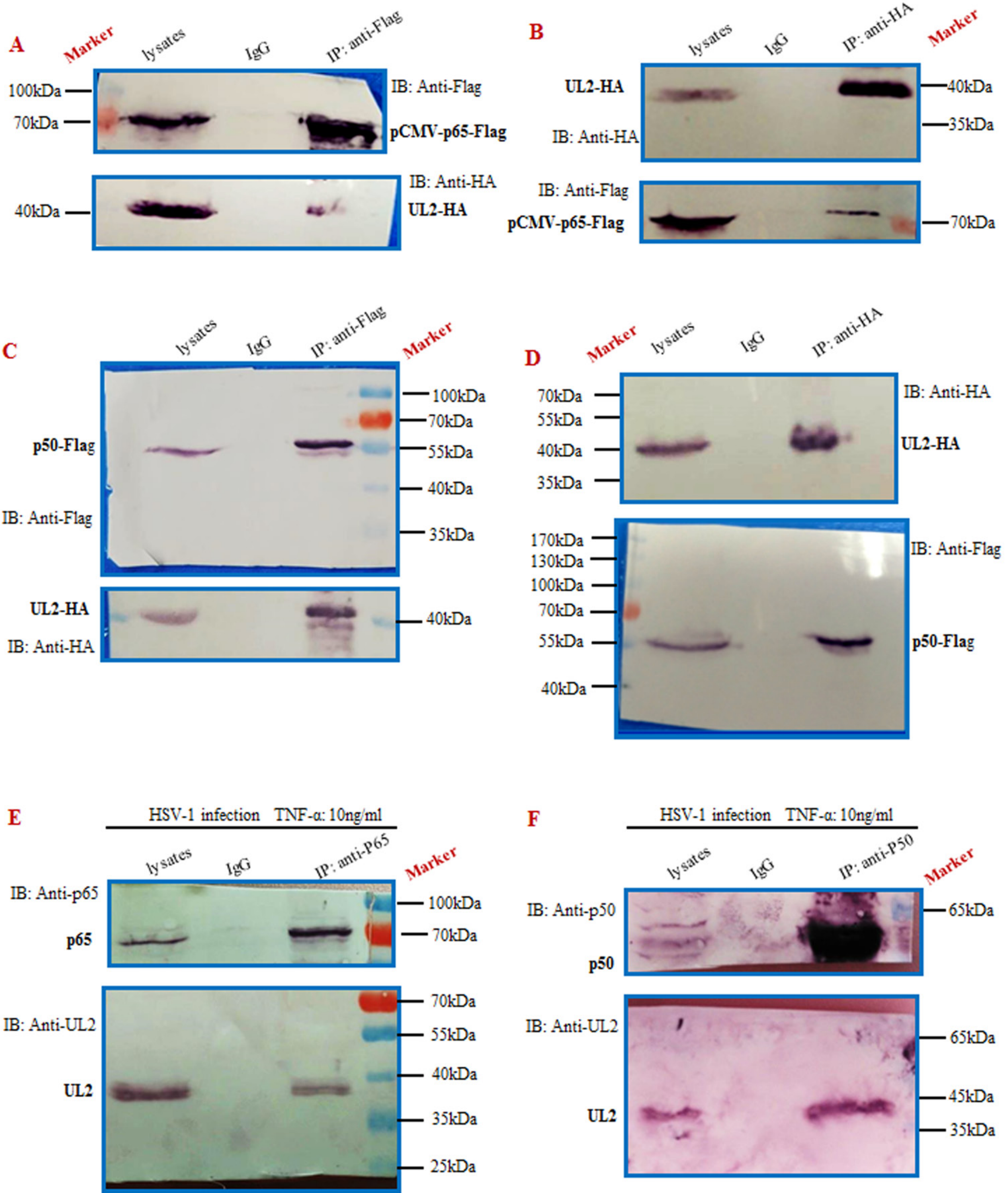

Original western blot results of Fig. 5

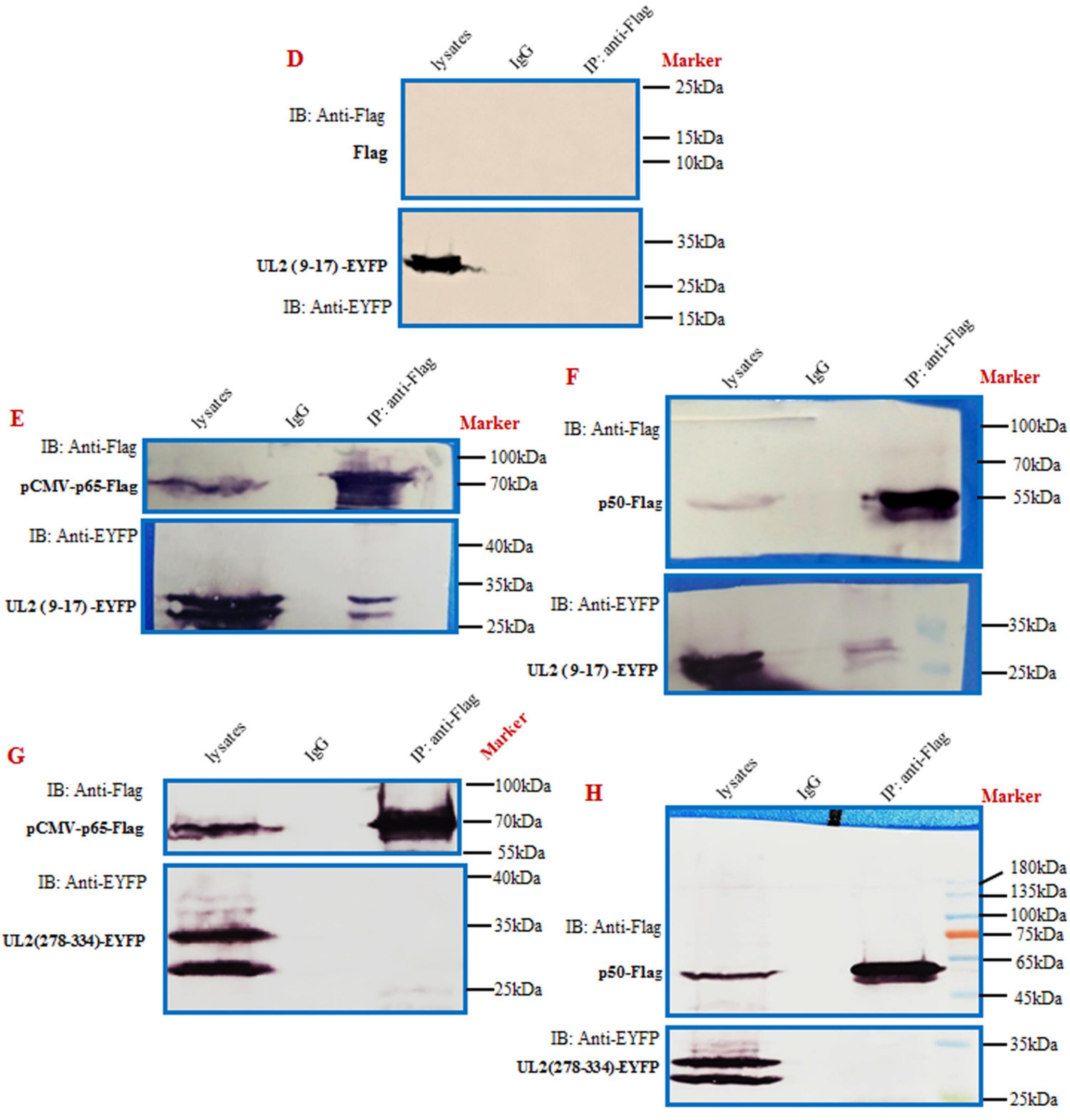

Original western blot results of Fig. 5

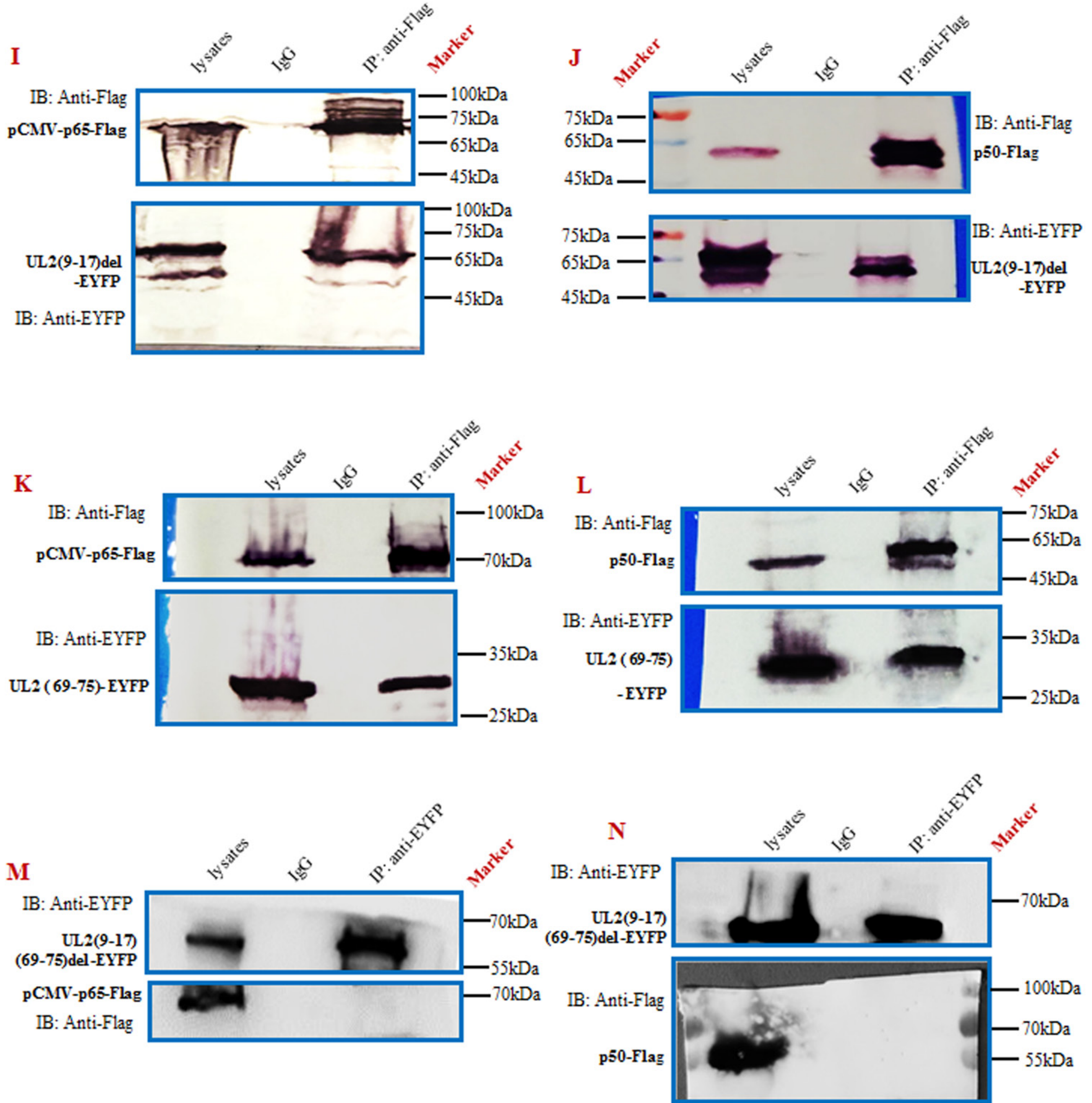

Original western blot results of Fig. 6

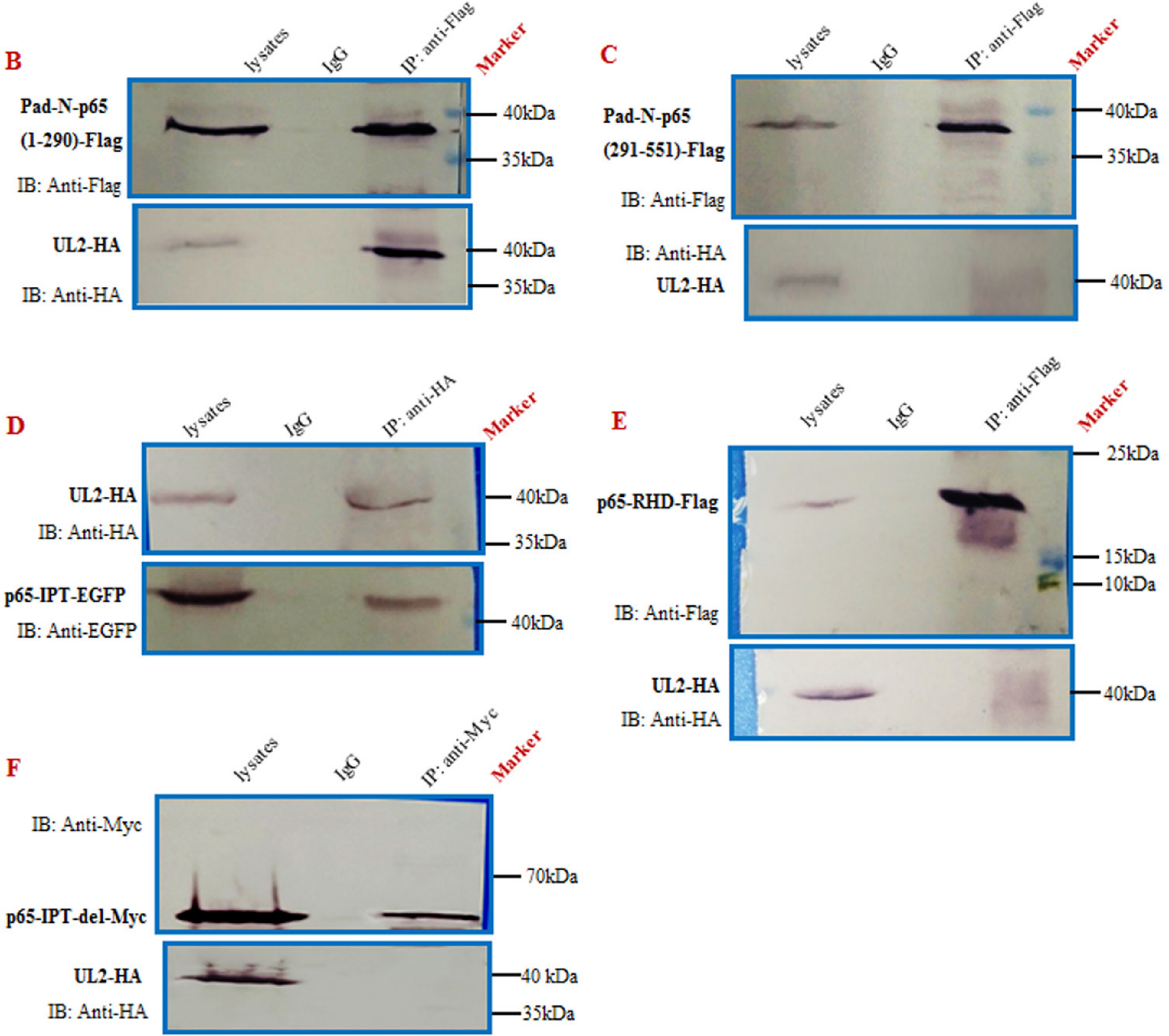

Original western blot results of Fig. 6

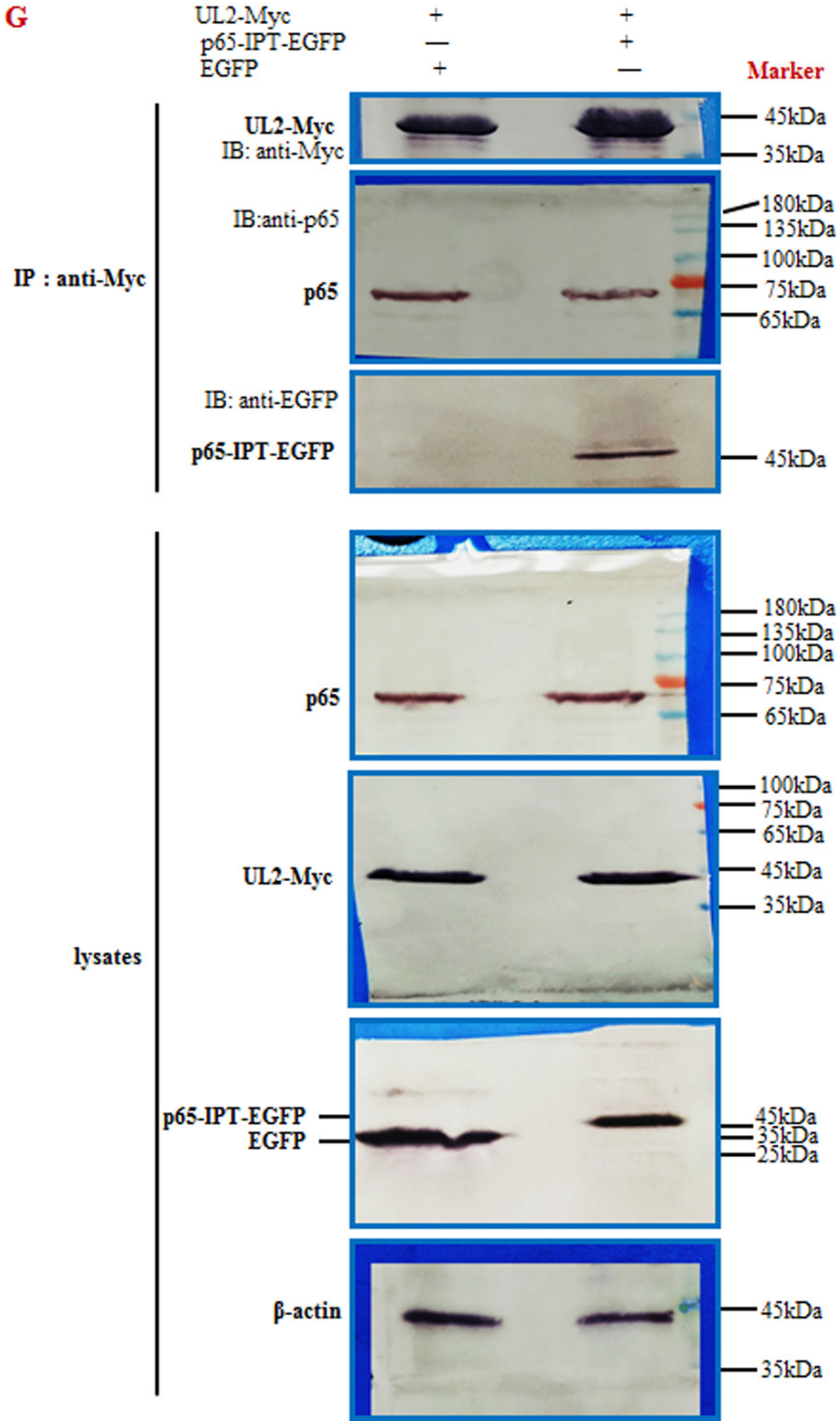

Original western blot results of Fig. 7

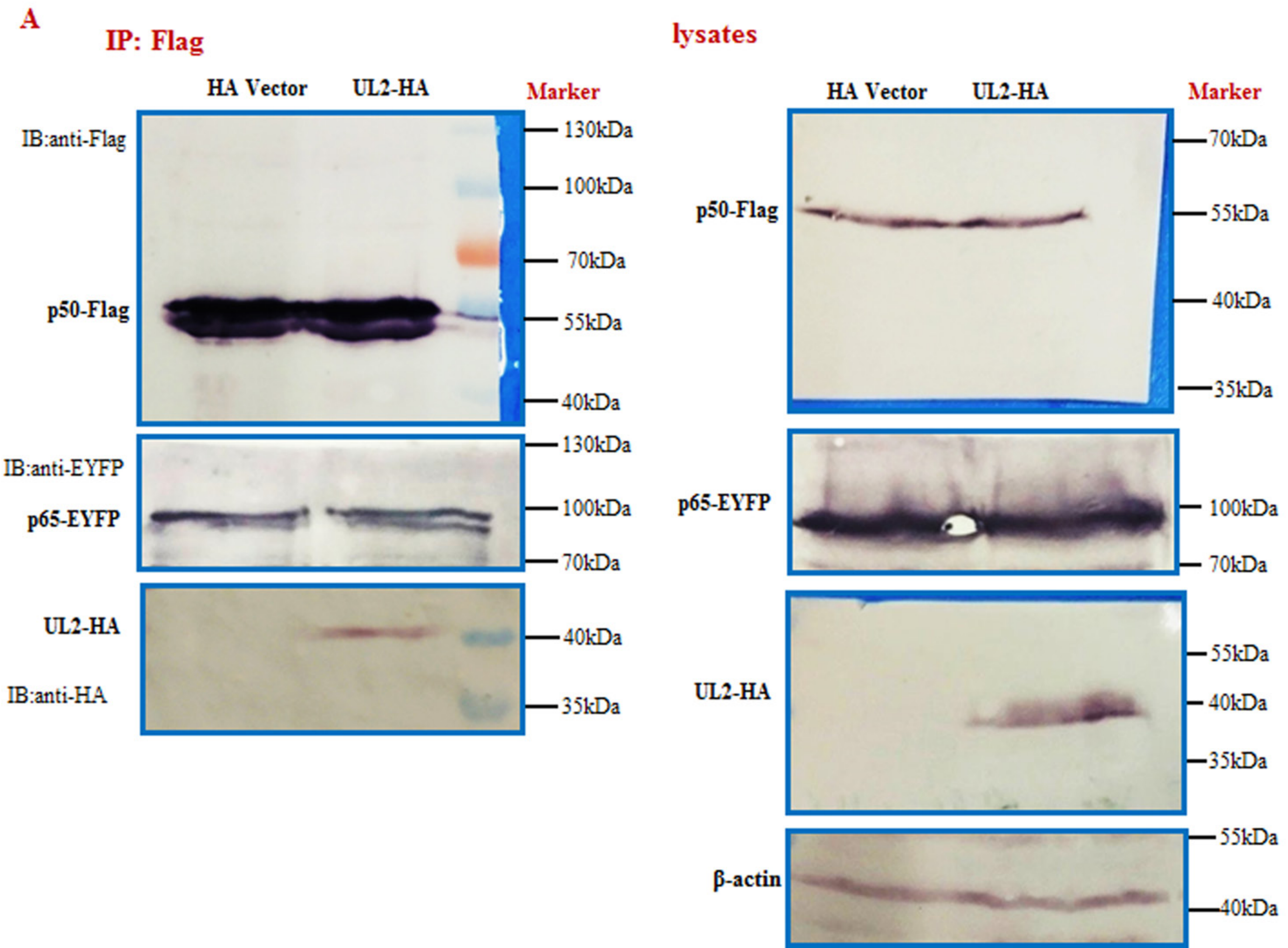

Original western blot results of Fig. 9

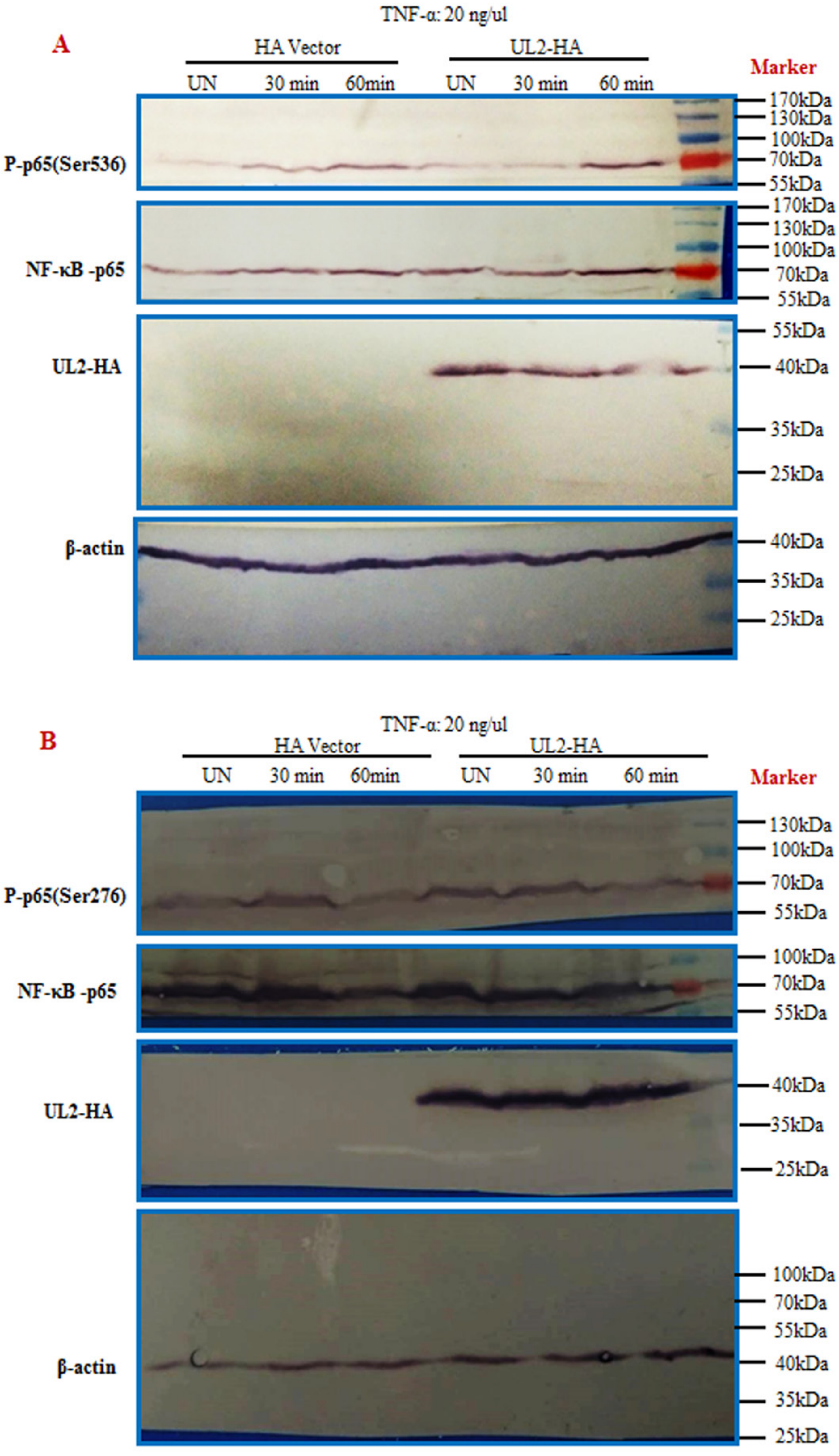

Supplement: Supplementary file 1 [file Data_Sheet_1.pdf]
